# The interplay of multimorbidity and depressive symptoms: mediation role of functional dependence

**DOI:** 10.1017/S2045796026100626

**Published:** 2026-04-07

**Authors:** Rui She, Haiyue Luo, Shanquan Chen, Fangfei Xiong, Karen P. Y. Liu, Marco Y. C. Pang

**Affiliations:** 1 Department of Rehabilitation Sciences, The Hong Kong Polytechnic University, Hong Kong, China; 2 Division of Community Medicine and Public Health Practice, School of Public Health, The University of Hong Kong, Hong Kong, China; 3 Independent Scholar, Puyang, China

**Keywords:** bi-directional association, depressive symptoms, functional dependence, mediation, multimorbidity

## Abstract

**Objective:**

Mental–physical multimorbidity is an emerging prevalent global health challenge. This study aims to examine reciprocal relationships between depressive symptoms and multimorbidity, with the mediation role of functional dependence in activities of daily living.

**Methods:**

Data were derived from the China Health and Retirement Longitudinal Study, which included 11,572 Chinese residents aged 45 years and older, surveyed in 2011, 2013, 2015 and 2018. Depressive symptoms were assessed using the Chinese version of the Center for Epidemiologic Studies Depression Scale (CESD-10) at baseline and each follow-up survey. Multimorbidity was operationalized as the condition count and the patterns identified via exploratory factor analysis. Four-wave cross-lagged panel models (CLPM) with bootstrapping were employed to estimate the path coefficients and the mediation effect of functional dependence.

**Results:**

Multimorbidity (cardiometabolic and respiratory-degenerative) and depressive symptoms exhibited bi-directional associations. Multimorbidity had a stronger impact on later depression (β: 0.042–0.130) than depression on multimorbidity (β: 0.005–0.064). Associations were stronger for respiratory-degenerative (β: 0.027–0.104) than cardiometabolic diseases (β: 0.005–0.065). Functional dependence partially mediated these links, with higher mediation for cardiometabolic (9–21%) than respiratory-degenerative diseases (4-6%). Additionally, some sex- and age-specific differences were identified in these dynamic associations.

**Conclusions:**

The study revealed bi-directional links between multimorbidity and depressive symptoms among Chinese adults. Functional dependence was a significant pathway in the cycle of multimorbidity and depressive symptoms, especially for cardiometabolic diseases. These insights suggest that interventions aimed at preventing functional dependence may be beneficial in mitigating the risk of coexisting mental and physical disorders.

## Introduction

Multimorbidity, the co-occurrence of two or more chronic diseases, is a prevalent global challenge, especially among older adults (e.g., 48% in aged 59–73 years and 67% in aged ≥74 years) (Ho *et al.*, 2022). Regarding multiple combinations of multimorbidity, the co-existence of mental and physical disorders is particularly noteworthy due to their synergistic impact on health outcomes (Moussavi *et al.*, 2007), including a 11.4-year reduction in life expectancy than the general populations and more than doubled mortality rates compared to individuals with mental-only or physical-only chronic conditions (Momen *et al.*, 2022). The complex multimorbidity pattern is expected to escalate, driven largely by population aging, increased life expectancy, and modern lifestyle behaviours (Diederichs *et al.*, 2011).

Existing segmented evidence indicates a bi-directional association between mental and physical disorders. Mental disorders, particularly depression, demonstrated a dose–response relationship with the number of co-occurring physical disorders (Barnett *et al.*, 2012). A meta-analysis indicated that individuals with multimorbidity had twice the risk of depression compared to those without multimorbidity (Read *et al.*, 2017). Conversely, depression was associated with increased risk of various chronic diseases, such as cardiovascular diseases and metabolic syndrome (Pan *et al.*, 2011; Vancampfort *et al.*, 2015, 2016). A multinational study suggested that depression was associated with 3.3 times higher odds of physical multimorbidity, particularly in China, where the odds ratio was 8.8 (Stubbs *et al.*, 2017). However, longitudinal studies exploring this hypothesis are scarce and often limited by traditional regression methods or short timeframes (Houlihan *et al.*, 2017; Read *et al.*, 2017; Ye *et al.*, 2022; Pengpid *et al.*, 2023; Ziwei *et al.*, 2023). Moreover, multimorbidity, often operationalized as the condition count in literature, may be insufficient to reflect the heterogeneity and interaction of chronic diseases comprising multimorbidity. A more detailed examination of specific multimorbidity patterns, which capture the non-random co-occurrence of diseases, could offer more nuanced insights into the potential pathological mechanisms of disease clusters and prevention and management of these intertwined health issues (She *et al.*, 2019).

Elucidating the mechanisms through which depression and physical diseases are linked is an important next step in addressing mental–physical multimorbidity. Increasing evidence suggests that functional dependence might mediate the bi-directional association between multimorbidity and depressive symptoms. Functional dependence in activities of daily living (ADL) is recognized as a pivotal determinant in the onset and progression of both mental and physical diseases (Lin and Wu, 2011; Xie *et al.*, 2018). Individuals with multiple chronic conditions tend to have inflammation and molecular damage, which decrease their physiological reserves and elevate their risk of ADL impairment (Read *et al.*, 2017; Pengpid *et al.*, 2023). This decline in functional independence can, in turn, engender negative emotions or feelings of worthlessness, increasing the susceptibility to depression (Bruce, 2001). Conversely, depressive symptoms can compromise individuals’ functional capacity and precipitate a decline in ADLs, potentially due to reduced motivation in maintaining social activities and impeded access to essential support networks (Kazama *et al.*, 2011). Chronic illnesses, such as cerebrovascular disease and cognitive impairment, are primary contributors to functional disability (Sousa *et al.*, 2009; Feng *et al.*, 2013; Su *et al.*, 2016; Hou *et al.*, 2018). Although several studies have explored the mediation effect of functional dependence in the link between multimorbidity and depressive symptoms (Jiang *et al.*, 2020; Leites de Souza Steffen *et al.*, 2020; Yang and Li, 2020; Ansari *et al.*, 2022; Smith *et al.*, 2022; Hu *et al.*, 2024), most have employed a cross-sectional approach and simply quantified multimorbidity as condition count, without examining the mutual mediation role of functional dependence in the interplay between multimorbidity and depressive symptoms. Furthermore, potential sex and age differences necessitate a stratified examination of these dynamic pathways. Epidemiological evidence shows that women have a higher prevalence of depressive symptoms and report greater functional limitations, while the burden of multimorbidity also varies by sex and increases with age (Ho *et al.*, 2022). Several cross-sectional studies suggested that the positive association between multimorbidity and mental distress was stronger for female than male adults (Jiao *et al.*, 2020; Lin *et al.*, 2021). These disparities may arise from a complex interplay of biological (e.g., hormonal, genetic), psychological (e.g., coping styles), and social factors (e.g., gendered caregiving roles, help-seeking behaviors). Consequently, the strength of the bidirectional associations and the mediating role of functional dependence may differ by sex and age. Exploring these differences is crucial for developing tailored and effective interventions.

This study, utilizing four waves of data spreading 8 years from a nationally representative sample of Chinese middle-aged to older adults, aimed to investigate the reciprocal relationship between depressive symptoms and multimorbidity as well as the mediation role of functional dependence in ADL. The research is guided by three key questions: (1) Are there bi-directional associations between multimorbidity and depressive symptoms and do these associations vary by different multimorbidity measures (condition count and patterns)? (2) To what extent does functional dependence in ADLs explain the bi-directional associations between multimorbidity and depressive symptoms? (3) Are there sex and age differences in the mediation effect of functional dependence within these bi-directional associations?

## Methods

### Study design and participants

Data were derived from the China Health and Retirement Longitudinal Study (CHARLS), which is a nationally representative and ongoing longitudinal study of Chinese residents aged 45 years and older. Participants were randomly recruited from 150 counties or districts and 450 villages or urban communities across 28 provinces (Zhao *et al.*, 2012). The baseline survey was conducted in 2011 and involved 17,708 respondents (response rate 80.5%), with biennial follow-ups in 2013, 2015 and 2018 (follow-up rate range: 85–91%). To exclude the potential confounding effect of the COVID-19 pandemic on participants’ mental and physical health (Shanbehzadeh *et al.*, 2021), the 2020 wave data were not used in this study. As the Supplementary Figure S1 shows, a total of 11,572 individuals who were 45 years or older and participated in all four waves of interviews were included in the final analysis.

### Ethical considerations

The ethical approval for the CHARLS study was granted by the Biomedical Ethics Committee of Peking University (IRB00001052-11015), and written informed consent was obtained from all participants.

### Measurements

#### Multimorbidity

A total of 13 chronic conditions across different time points were defined consistently, using data collected at baseline and follow-up surveys. Specifically, hypertension, diabetes, dyslipidaemia, heart disease, stroke, chronic lung disease (i.e., chronic bronchitis and emphysema), digestive disease, kidney disease, memory‐related disease, arthritis and asthma were ascertained based on self-reported physician diagnosis or if the person reported being receiving corresponding treatment (e.g., medication or surgery). Vision impairment was defined by self-reported poor eyesight for seeing things both at a distance and up close. Hearing impairment was evaluated by rating their hearing status as poor or very poor, regardless of hearing aid use.

#### Depressive symptoms

The Chinese version of the 10‐item Center for Epidemiologic Studies Depression Scale (CESD-10) was administered at baseline and each follow-up, which has been well-validated as a reliable tool for detecting depression in Chinese adults (Jiang *et al.*, 2019). The respondents were asked to rate the degree of depressed mood and positive affect felt over the past week, with each item rated on a four‐point scale. Its summative scores range from 0 to 30, with higher scores indicating more severe depressive symptoms.

#### Functional dependence in ADLs

The levels of difficulty in performing six activities of daily living were evaluated: dressing, bathing, eating, transferring (getting in or out of bed), toileting and continence (Katz, 1983). Participants were scored 1 point for each activity they had some difficulty performing or were unable to carry out. The total scores range from 0 to 6, with higher values denoting greater levels of functional dependence.

#### Covariates

Socio-demographic covariates included age, sex, residency (rural or urban), marital status, educational levels, coverage of public health insurance, number of children and retirement status (yes or no). Lifestyle characteristics included the number of days per week that participants engaged in any form of physical activity, current smoking status (yes or no) and alcohol consumption in the last year. The levels of social participation were evaluated based on the number of involved activities within the past month: socializing with friends, playing games, attending sporting events or clubs, participating in community organizations, engaging in voluntary or charity work, attending educational or training courses and maintaining contact with children.

#### Statistical analysis

Descriptive data were presented as mean (standard deviation, SD) for continuous variables and frequency (percentage) for categorical variables.

Multimorbidity was calculated by counting the number of chronic diseases at each wave. To identify patterns of multimorbidity, exploratory factor analysis (EFA) was performed using the baseline data. This analysis utilized a tetrachoric correlation matrix suitable for binary indicators and the principal component method, with oblique (Oblimin) rotation to enhance the interpretability of the factors (Mislevy, 1985). The optimal number of factors retained was determined based on eigenvalues, the scree plot and interpretability (Prados-Torres *et al.*, 2012). A specific condition was selected to characterize a pattern if its corresponding factor loading was above 0.25, which indicates a strong association of the condition with its disease pattern (Schäfer *et al.*, 2010). Standardized composite factor scores were calculated for each factor, with a higher score suggesting a greater number of conditions belonging to a specific pattern. During the follow-up surveys, factor scores for each pattern were estimated using the baseline multimorbidity patterns’ factor loadings to capture the dynamic changes in multimorbidity patterns over time (Shi *et al.*, 2022).

**Figure 1.**
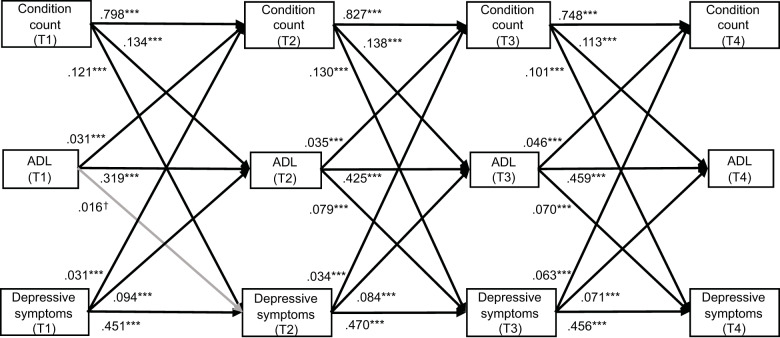
Cross-lagged panel models for the mediation effects of functional dependence in activities in daily living (ADL) in the reciprocal association between condition count and depressive symptoms from 2011 to 2018 (n=11,572). Note: Solid lines represent the significance of the structural path (p< 0.05) while dash lines represent non-significant paths, and grey lines represent marginal significant paths (0.05< p <0.10). Standardized coefficients were shown. For simplicity, background covariates of outcomes and correlation paths are not presented. ***p<0.001, **p<0.01, *p<0.05, †0.05<*p*<0.10.

**Figure 2.**
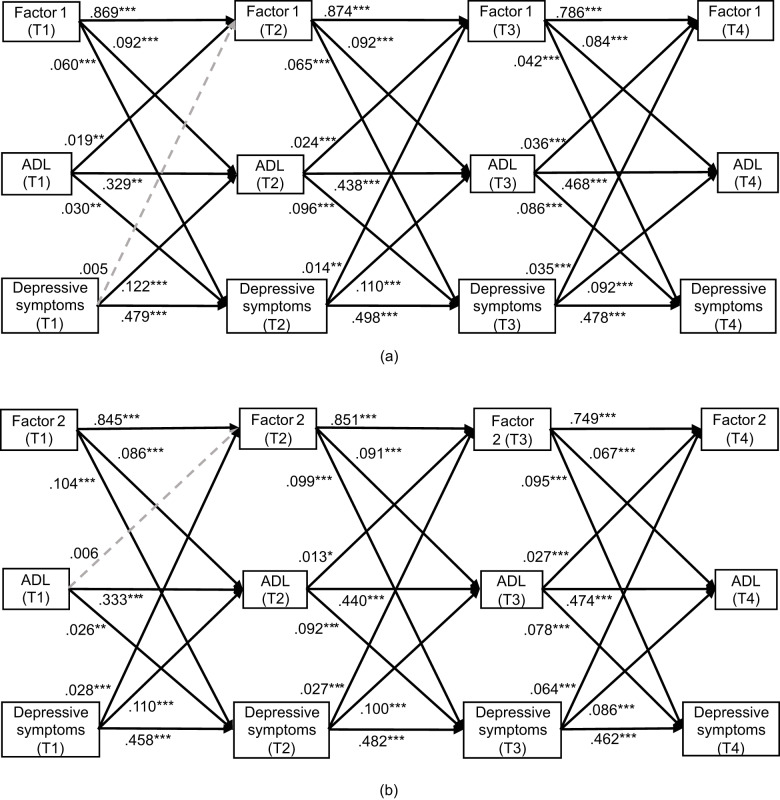
Cross-lagged panel models for the mediation effects of functional dependence in activities in daily living (ADL) in the reciprocal association between multimorbidity patterns and depressive symptoms from 2011 to 2018 (n=11,572). Note: Factor 1, cardiometabolic disease pattern; Factor 2, respiratory-degenerative disease pattern. Solid lines represent the significance of the structural path (p< 0.05) while dash lines represent non-significant paths, and grey lines represent marginal significant paths (0.05<p<0.10). Standardized coefficients were shown. For simplicity, background covariates of outcomes and correlation paths are not presented. ***p<0.001, **p<0.01, *p<0.05, †0.05<*p*<0.10.

We examined the mediation relationships of functional dependence in the reciprocal associations between multimorbidity and depressive symptoms, by using cross-lagged panel models (CLPM) which is a widely used and sophisticated approach for evaluating temporal and mediation relationships in longitudinal data (Preacher, 2015; Wu *et al.*, 2018). In this study, CLPM models specified bi-directional pathways among multimorbidity, functional dependence in ADL, and depressive symptoms, predicting the subsequent time points (Figures 1–2). Cross-lagged effect size was categorized as small (0.03), medium (0.07), or large (0.12) in magnitude (Orth *et al.*, 2022). The goodness-of-fit of CLPM models was evaluated using the following criteria: root mean square error of approximation (RMSEA) ≤0.08, comparative fit index (CFI) ≥ 0.90, and standardized root mean squared residual (SRMR) ≤ 0.08 (Byrne, 2013). Missing data were handled using the full information maximum likelihood (FIML) method, which could produce less biased and more reliable estimates compared with conventional methods of dealing with missing data (Enders and Bandalos, 2001; Baraldi and Enders, 2010). Bootstrapping (*n* = 2000) was applied to estimate the standardized path coefficients (*β*) and indirect effects of functional dependence in the reciprocal associations between multimorbidity and depressive symptoms. The proportion mediated (indirect effects/total effects) was calculated to quantify the mediation effect size (Cole and Maxwell, 2003).

Finally, to consider sex differences, CLPM models were estimated separately for males and females. Additionally, subgroup analyses were conducted by stratifying participants into two age groups (≤60 years vs. > 60 years). Differences in individual indirect effect paths were examined by Wald tests. A nonsignificant Wald chi-square test of difference suggests that the magnitude of indirect path is invariant for two groups, whereas a significant test indicates that the path is variant.

Data analyses were performed using SPSS 27, STATA 17.0, and Mplus 8.0, with a two-tailed *P-*value of < 0.05 considered statistically significant.

#### Sensitivity analyses

To ensure the robustness of our findings, we conducted multiple sensitivity analyses. First, we repeated our analyses including participants with baseline and at least one follow-up assessment to evaluate potential attrition effects (Supplementary Table S1, Figures S2–S4). Second, we excluded memory-related diseases from the multimorbidity measure to focus on physical conditions only (Supplementary Table S2, Figures S5–S7). Results from both sensitivity analyses remained consistent with our primary findings, confirming the robustness of our conclusions.

## Results

### Descriptive results

#### Characteristics of the participants

At baseline, the mean (SD) age of the participants was 58.2 (8.83) years. The majority of participants were female (52.8%), married/partnered (83.2%), lived in urban areas (82.0%), and covered by public health insurance (92.8%). Of all participants, 68.0% were current smokers, 66.3% had alcohol drinking in the past year, and 33.6% had daily physical activity. The most prevalent chronic conditions among the participants were arthritis (37.6%), followed by visual impairment (35.4%) and hypertension (26.4%). The mean score (SD) for depressive symptoms assessed by CESD-10 was 8.39 (6.29) (Table 1).

**Table 1.** Baseline characteristics of study participants in the 2011 wave survey (*N* = 11,572)

Characteristics	*N* (%)/Mean土SD
**Age (year)**	58.2 ± 8.83
**Sex**	
Male	5457 (47.2)
Female	6115 (52.8)
**Educational levels**	
No Formal education	3224 (27.9)
Lower than elementary	2144 (18.5)
Elementary	2509 (21.7)
Mid, high, vocational school or tertiary	3695 (31.9)
**Residential area**	
Rural	2066 (17.9)
Urban	9493 (82.0)
Missing	13 (0.1)
**Public health insurance**	
Yes	10 742 (92.8)
No	738 (6.4)
Missing	92 (0.8)
**Marital status**	
Married or partnered	9624 (83.2)
Separated or divorced, and single	896 (7.7)
Widowed	1042 (9.0)
Missing	10 (0.1)
**Number of children**	
0 – 1	2098 (18.1)
2	4190 (36.2)
≥3	5274 (45.6)
Missing	10 (0.1)
**Retirement status**	
Yes	3389 (29.3)
No	7969 (68.9)
Missing	214 (1.8)
**Number of days for physical activity per week**	
None	7364 (63.6)
1−6 days	315 (2.7)
Daily	3893 (33.6)
**Current smoker**	
Yes	7874 (68.0)
No	3326 (28.7)
Missing	372 (3.2)
**Alcohol consumption in the last year**	
Yes	7673 (66.3)
No	3843 (33.2)
Missing	56 (0.5)
**Social participation in the past months**	
None	750 (6.5)
1 activity	7439 (64.3)
2 activities	2657 (23.0)
≥3 activities	689 (5.9)
Missing	37 (0.3)
**Chronic physical conditions**	
Arthritis	4325 (37.6)
Visual impairment	3837 (35.4)
Hypertension	3025 (26.4)
Digestive diseases	3000 (26.1)
Hearing impairment	1580 (13.7)
Heart diseases	1486 (13.0)
Chronic lung diseases	1259 (11.0)
Dyslipidemia	1210 (10.7)
Kidney diseases	777 (6.8)
Diabetes	688 (6.0)
Asthma	480 (4.2)
Stroke	286 (2.5)
Memory diseases	180 (1.6)
**Depressive symptoms (CESD − 10)**	8.39 ± 6.29

Note: SD, Standard deviation. Number of missing values for Arthritis: 80 (0.7%); Visual impairment 741 (6.4%); Hypertension 114 (1.0%); Digestive diseases 87 (0.8%); Hearing impairment 58 (0.5%); Heart diseases 121 (1.0%); Chronic lung diseases 99 (0.9%); Dyslipidemia 283 (2.4%); Kidney diseases 131 (1.1%); Diabetes 157 (1.4%); Asthma 101 (0.9%); Stroke 83 (0.7%); Memory diseases 94 (0.8%). The prevalence of a chronic condition was calculated by dividing the total number of participants having a specific disease by the number of participants without missing data on the corresponding condition.

#### Patterns of multimorbidity

The Kaiser–Meyer–Olkin measure of 0.72 indicated a moderate sampling adequacy for performing a factor analysis. Two distinct multimorbidity patterns were identified, collectively accounting for 95% of the total variance (Table 2). Factor 1 was characterized by cardiometabolic diseases, including dyslipidaemia, hypertension, diabetes, heart diseases, stroke and memory diseases. Factor 2 was primarily characterized by respiratory-degenerative ailments, such as lung diseases, arthritis, digestive diseases, hearing impairment, kidney diseases and vision impairment.

**Table 2.** Rotated factor loadings for each of the 13 chronic diseases by disease patterns from factor analysis using the baseline data

Chronic diseases	Cardiometabolic diseases	Respiratory-degenerative diseases
Dyslipidemia	**0.69**	−0.09
Hypertension	**0.64**	−0.04
Diabetes	**0.58**	−0.07
Stroke	**0.53**	0.02
Heart diseases	**0.53**	0.23
Memory diseases	**0.39**	0.21
Chronic lung diseases	−0.05	**0.77**
Asthma	−0.01	**0.73**
Arthritis	0.19	**0.34**
Digestive diseases	0.11	**0.32**
Hearing impairment	0.14	**0.29**
Kidney diseases	0.15	**0.28**
Vision impairment	0.15	**0.25**
**Eigenvalue**	2.10	1.68
**Proportion explained**	63.3%	31.8%

#### Correlations between studied variables

Supplementary Table S3 reveals positive and consistent correlations among multimorbidity measures, functional dependence in ADL and depressive symptoms across all four waves (all *P* < 0.01).

#### CLPM results

Three individual CLPM models were fitted for each multimorbidity measure, adjusting for sociodemographic and lifestyle covariates. The model fit indices, presented in Supplementary Table S4, supported the validity of CLPM models (CFI > 0.90, RMSEA < 0.080 and SMRA < 0.08).

#### Condition count model

As shown in Fig. 1, significant autoregressive paths suggested the stability of the number of chronic conditions (range for *β*: 0.748–0.827; all *P* < 0.001), functional dependence in ADL (range for *β*: 0.319–0.459; all *P* < 0.001), and depressive symptoms (range for *β*: 0.451–0.470; all *P* < 0.001) over time, indicating that these variables were significantly predictive of their subsequent scores at followed-up assessment. All the cross-lagged paths remained significant, except the relationship between functional dependence at T1 and depressive symptoms at T2, which was marginally significant (0.05 < *P* < 0.10). A higher number of chronic conditions predicted greater functional dependence in ADL (range for *β*: 0.113–0.138; all *P* < 0.001) and depressive symptoms (range for *β*: 0.101–0.130; all *P* < 0.001) at subsequent assessment. Additionally, depressive symptoms were predictive of greater functional dependence (range for *β:* 0.071–0.094; all *P* < 0.001) and an accumulation of chronic conditions (range for *β:* 0.031–0.063; all *P* < 0.001) in the following wave.

The bootstrap mediation analysis indicated that functional dependence partially mediated the prospective associations between the accumulation of multimorbidity and depressive symptoms, and vice versa (Table 3). Specifically, functional dependence accounted for 6.2% of the association between the number of chronic conditions at T1 and depressive symptoms at T3 (indirect effect: *β* = 0.011, *P* < 0.05) and 6.3% of the association between condition count at T2 and depressive symptoms at T4 (indirect effect: *β* = 0.010, *P* < 0.05). On the other hand, functional dependence mediated 7.4% of the association between depressive symptoms at T1 and accumulation of chronic conditions at T3 (indirect effect: *β* = 0.003, *P* < 0.05). The mediation effect from T2 to T4 was similar (proportion mediated: 6.6 %).

**Table 3.** Parameter estimates on the associations of multimorbidity, functional dependence in ADL and depressive symptoms from the cross-lagged panel models with mediation by sex

Paths	Total sample (*n* = 11,572)		Male (*n* = 5457)		Female (*n* = 6115)		Wald test for difference
	Standardized indirect effect *β* (95% CI)	%	Standardized indirect effect *β* (95% CI)	%	Standardized indirect effect *β* (95% CI)	%	*P*-value
**Chronic condition count**							
Depressive symptoms (T1) →ADL (T2)→Condition count (T3)	**0.003**	7.4	**0.002**	5.3	**0.004**	9.5	0.244
	**[0.002, 0.005]**		**[0.001, 0.004]**		**[0.002, 0.006]**		
Condition count (T1)→ADL (T2)→Depressive symptoms (T3)	**0.011**	6.2	**0.014**	8.2	**0.008**	5.2	0.087
	**[0.008, 0.013]**		**[0.010,0.019]**		**[0.005, 0.012]**		
Depressive symptoms (T2)→ADL (T3)→Condition count (T4)	**0.004**	6.6	**0.003**	6.2	**0.004**	6.5	0.693
	**[0.002, 0.005]**		**[0.001, 0.005]**		**[0.002, 0.006]**		
Condition count (T2) →ADL (T3)→Depressive symptoms (T4)	**0.010**	6.3	**0.008**	4.8	**0.010**	7.4	0.282
	**[0.007, 0.013]**		**[0.004, 0.012]**		**[0.005, 0.014]**		
**Cardiometabolic disease pattern (Factor 1)**							
Depressive symptoms (T1)→ADL (T2)→Factor 1 score (T3)	**0.003**	21.4	**0.002**	18.5	**0.003**	24.7	0.427
	**[0.001, 0.004]**		**[0.000, 0.004]**		**[0.002, 0.005]**		
Factor 1 score (T1)→ADL (T2)→Depressive symptoms (T3)	**0.009**	9.3	**0.012**	13.4	**0.007**	7.8	0.080
	**[0.006, 0.012]**		**[0.008, 0.016]**		**[0.004, 0.010]**		
Depressive symptoms (T2)→ADL (T3)→Factor 1 score (T4)	**0.004**	12.2	**0.003**	12.4	**0.004**	12.1	0.473
	**[0.002, 0.006]**		**[0.001, 0.005]**		**[0.002, 0.007]**		
Factor 1 score (T2)→ADL (T3)→Depressive symptoms (T4)	**0.008**	10.5	**0.008**	9.4	**0.008**	12.2	0.745
	**[0.005, 0.010]**		**[0.004, 0.011]**		**[0.005, 0.011]**		
**Respiratory-degenerative diseases pattern (Factor 2)**							
Depressive symptoms (T1)→ADL (T2)→Factor 2 score (T3)	**0.001**	3.8	0.000	0.4	**0.002**	5.2	0.231
	**[0.000, 0.003]**		[−0.001, 0.002]		**[0.000, 0.004]**		
Factor 2 score (T1)→ADL (T2)→Depressive symptoms (T3)	**0.008**	5.6	**0.010**	6.5	**0.007**	4.8	0.420
	**[0.006, 0.010]**		**[0.005, 0.015]**		**[0.003, 0.010]**		
Depressive symptoms (T2)→ADL (T3)→Factor 2 score (T4)	**0.003**	5.0	**0.002**	3.9	**0.003**	5.6	0.463
	**[0.001, 0.004]**		**[0.000, 0.004]**		**[0.001, 0.005]**		
Factor 2 score (T2)→ADL (T3)→Depressive symptoms (T4)	**0.007**	5.3	**0.005**	3.6	**0.008**	6.3	0.058
	**[0.005, 0.009]**		**[0.002, 0.009]**		**[0.004, 0.012]**		

Note: CI, confidence interval; %, Proportion mediated by functional dependence in ADL; ADL, functional dependence in activities in daily living (ADL). Significant indirect effects were in bold.

#### Multimorbidity pattern models

Similar bi-directional associations among multimorbidity patterns, functional dependence, and depressive symptoms were observed in the two models examining multimorbidity patterns. As depicted in Fig. 2a, accumulated cardiometabolic diseases (factor 1) predicted the subsequent decline in functional capacity (range for *β:* 0.084–0.092; all *P <* 0.001) and increased depressive symptoms (range for *β:* 0.042–0.065; all *P <* 0.001). Furthermore, depressive symptoms predicted higher levels of functional dependence (range for *β:* 0.092–0.122; all *P <* 0.001) and accumulation of cardiometabolic diseases in the subsequent wave (T2→T3: *β* = 0.014; T3→T4: *β* = 0.035), whereas depressive symptoms at T1 failed to predict factor 1 at T2. Moreover, participants with more respiratory-degenerative diseases were more likely to have depressive symptoms (range for *β:* 0.095–0.104; all *P <* 0.001) while depressive symptoms predicted the accumulation of respiratory-degenerative diseases in the subsequent wave (range for *β:* 0.027–0.064; all *P <* 0.001) (Fig. 2b).

The mediation effect of functional dependence remained significant in the reciprocal associations between specific multimorbidity patterns and depressive symptoms. Functional dependence mediated a larger proportion of the total association between cardiometabolic diseases and depressive symptoms (∼9–11%) compared to that of the association between respiratory-degenerative diseases and depressive symptoms (∼5–6%). Regarding the association between depressive symptoms and multimorbidity patterns, the mediated proportion of functional dependence in ADL was higher for cardiometabolic diseases (∼12–21%) than that for respiratory-degenerative diseases (∼4–5%) (Table 3).

#### Multigroup analyses by sex and age

In both male and female subgroups, functional dependence was as a partial mediator in the bi-directional associations between multimorbidity measures and depressive symptoms, with the exception of the indirect path from depressive symptoms at T1 to respiratory-degenerative disease pattern at T3 via functional dependence among males (Table 3). Furthermore, the Wald test indicated no significant sex differences in the mediation effect attributed to functional dependence (all *P* > 0.05).

Nevertheless, some divergences in path coefficients between sexes were observed (Supplementary Figures S8–S13). In the cardiovascular disease pattern model, more cross-lagged paths between depressive symptoms and cardiovascular diseases were non-significant in males than females. Furthermore, the cross-lagged paths between functional dependence and respiratory-degenerative diseases were exclusively significant among females.

Regarding age subgroups, functional dependence generally remained as a mediator in the bi-directional associations between multimorbidity measures and depressive symptoms, with no significant age differences in the mediation effect of functional dependence as indicated by the Wald test (all *P* > 0.05; Table 4). However, among participants aged > 60 years, the cross-lagged paths between functional dependence and respiratory-degenerative diseases were non-significant (Supplementary Figure S19). Consequently, functional dependence did not mediate the association between depressive symptoms and respiratory-degenerative diseases in this older subgroup. Detailed modelling results are presented in Supplementary Figures S14–S19.

**Table 4.** Parameter estimates on the associations of multimorbidity, functional dependence in ADL, and depressive symptoms from the cross-lagged panel models with mediation by age

	Age of 60 or less (*n* = 7341)		Beyond age of 60 (*n* = 4214)		Wald test for difference
Paths	Standardized indirect effect *β* (95% CI)	%	Standardized indirect effect *β* (95% CI)	%	*P*-value
**Chronic condition count**					
Depressive symptoms (T1) →ADL (T2)→Condition count (T3)	**0.004**	7.2	**0.003**	6.8	0.417
	**[0.002, 0.005]**		**[0.000, 0.005]**		
Condition count (T1)→ADL (T2)→Depressive symptoms (T3)	**0.010**	5.7	**0.009**	5.4	0.868
	**[0.006, 0.014]**		**[0.005, 0.014]**		
Depressive symptoms (T2)→ADL (T3)→Condition count (T4)	**0.004**	6.6	**0.004**	7.4	0.256
	**[0.003, 0.007]**		**[0.001, 0.007]**		
Condition count (T2) →ADL (T3)→Depressive symptoms (T4)	**0.009**	6.3	**0.009**	5.3	0.340
	**[0.005, 0.013]**		**[0.004, 0.013]**		
**Cardiometabolic disease pattern (Factor 1)**					
Depressive symptoms (T1)→ADL (T2)→Factor 1 score (T3)	**0.003**	14.0	**0.003**	8.5	0.426
	**[0.001, 0.004]**		**[0.000, 0.005]**		
Factor 1 score (T1)→ADL (T2)→Depressive symptoms (T3)	**0.009**	7.2	**0.007**	7.2	0.838
	**[0.005, 0.012]**		**[0.003, 0.011]**		
Depressive symptoms (T2)→ADL (T3)→Factor 1 score (T4)	**0.004**	10.1	**0.004**	16.1	0.169
	**[0.002, 0.006]**		**[0.002, 0.007]**		
Factor 1 score (T2)→ADL (T3)→Depressive symptoms (T4)	**0.007**	11.2	**0.007**	8.2	0.263
	**[0.004, 0.010]**		**[0.003, 0.011]**		
**Respiratory-degenerative diseases pattern (Factor 2)**					
Depressive symptoms (T1)→ADL (T2)→Factor 2 score (T3)	**0.002**	5.5	0.000	0.0	0.423
	**[0.000, 0.004]**		[−0.002, 0.002]		
Factor 2 score (T1)→ADL (T2)→Depressive symptoms (T3)	**0.006**	4.2	**0.008**	5.5	0.843
	**[0.003, 0.009]**		**[0.004, 0.012]**		
Depressive symptoms (T2)→ADL (T3)→Factor 2 score (T4)	**0.004**	6.4	0.002	3.3	0.162
	**[0.001, 0.006]**		[−0.001,0.004]		
Factor 2 score (T2)→ADL (T3)→Depressive symptoms (T4)	**0.007**	4.9	**0.006**	4.5	0.267
	**[0.003, 0.010]**		**[0.003, 0.010]**		

Note: CI, confidence interval; %, Proportion mediated by functional dependence in ADL; ADL, functional dependence in activities in daily living (ADL). Significant indirect effects were in bold.

## Discussion

By utilizing four-wave CLPM models, this longitudinal study firstly provided longitudinal evidence on the bi-directional associations between various multimorbidity measures-including condition count and specific patterns-and depressive symptoms over 8 years in a nationally representative cohort of middle-aged to older Chinese adults. Notably, multimorbidity showed a more pronounced cross-lagged effect on later depressive symptoms than the reverse, emphasizing the need to strengthen the proper management of chronic conditions for the prevention of deterioration or development of more chronic conditions, and meanwhile integrate mental health services into the management of chronic conditions. Additionally, we firstly found that functional dependence in ADL served as a partial mediator in the prospective association between the accumulation of multimorbidity and depressive symptoms, and vice versa, with a stronger mediation effect for cardiometabolic diseases. This study advances the understanding of the intricate dynamics between chronic conditions and psychological health. By elucidating the pathways underpinning their associations, the findings could inform the prevention and intervention of comorbid mental and physical health conditions.

Our findings of bi-directional associations between multimorbidity and depressive symptoms concur with previous studies investigating their unidirectional associations or using logistic regression models (Stubbs *et al.*, 2017; Ye *et al.*, 2022). The cycle between multimorbidity and depressive symptoms aligns with the Biopsychosocial model, which posits that the medical condition is determined by the interplay of biological, psychological and social factors (Engel, 1977; Schotte *et al.*, 2006). By using CLPM that investigated multimorbidity and depressive symptoms simultaneously and dynamically, our study found that the effect of multimorbidity on subsequent depressive symptoms was medium-to-large, while the reverse effect was small-to-medium. This underscores the considerable psychological burden of chronic conditions, possibly due to the common associated pain or fatigue (Campbell *et al.*, 2003). These findings appear to contrast with previous meta-analyses that indicated a doubled risk of depression in individuals with physical multimorbidity (Read *et al.*, 2017) and a tripled risk of physical multimorbidity among those with depression, compared to their counterparts (Stubbs *et al.*, 2017). Nonetheless, the heterogeneity in methodology and cross-sectional nature of included studies make it difficult to derive direct comparisons and illustrate the directionality. By analysing data from the same cohort, our study offers new and valid evidence on the directionality and magnitude of these associations.

Moreover, we contributed to the extant literature by clarifying the relationships between specific multimorbidity patterns and depressive symptoms. We found that the accumulation of respiratory-degenerative diseases demonstrated a stronger bi-directional association with depressive symptoms, as indicated by the magnitude of cross-lagged effects, compared to cardiometabolic diseases. These two multimorbidity patterns, identified in our study, were identical to those identified in prior studies of older adults in China (Gu *et al.*, 2018; She *et al.*, 2019). This distinction may be attributed to the heavier psychological toll exerted by respiratory diseases and arthritis compared to cardiometabolic diseases, involving mechanisms such as chronic inflammation, breathing discomfort and life disruption caused by these diseases (Penninx *et al.*, 1996; Siboni *et al.*, 2019; Han *et al.*, 2021). Conversely, managing cardiometabolic diseases, such as diabetes, often entails lifestyle modifications that can be empowering and provide a sense of control, potentially buffering the psychological distress associated with chronic illness (Warren *et al.*, 2013). Mental health support and services should be integrated especially into the management of respiratory-degenerative diseases.

The mediation analysis illuminated the significance of functional dependence in explaining the bi-directional relationship between multimorbidity and depressive symptoms over time. The accumulation of chronic conditions not only directly elevated the level of depressive symptoms but also indirectly through changes in functional dependence. On the other hand, depressive symptoms predicted the progression of functional dependence, which in turn led to the accumulation of chronic conditions. This highlights the pivotal role of functional disability as a critical pathway in the cycle between mental and physical multimorbidity. Interventions aimed at preventing ADL disability may be effective in mitigating the co-occurrence of mental and physical health issues. Notably, functional dependence accounted for a larger mediation effect (9–21%) in the relationship between depressive symptoms and cardiometabolic diseases over respiratory-degenerative diseases (4–6%). This suggests that functional dependence may be a more prominent pathway in the context of cardiometabolic diseases. Nevertheless, the partial mediation indicates the existence of other plausible mediators contributing to the reciprocal associations between chronic conditions and depressive symptoms. Previous research has proposed that poor sleep quality, chronic pain, and social support could mediate the multimorbidity-depression relationship (Zis *et al.*, 2017; Demirer *et al.*, 2021; Ansari *et al.*, 2022), which should be verified in future longitudinal studies to fully unravel the underlying mechanisms.

Sex-specific and age-specific analyses indicated a generally consistent mediation role of functional dependence in the bi-directional association between multimorbidity and depressive symptoms across subgroups, with similar magnitudes of indirect effects. However, a few discrepancies emerged. For example, the association between functional dependence and respiratory-degenerative diseases was not statistically significant in males, and consequently, the indirect path from depressive symptoms at T1 to respiratory-degenerative diseases at T3 via functional dependence was not significant among men. Women tend to report higher levels of functional limitations and more severe manifestations of arthritis and respiratory diseases, whereas men may underreport health issues due to cultural norms and societal expectations (Orellano-Colón *et al.*, 2021; Hu *et al.*, 2024). This might partly explain the stronger association between functional dependence and respiratory-degenerative diseases among females. In addition, the prospective association between depressive symptoms and the accumulation of cardiometabolic diseases was more evident in females. Similarly, a previous cross-sectional study among older adults in Australia and the United States found that the association between depressive symptoms and metabolic syndrome was exclusively significant in women, which might be attributed to the sex-specific pathophysiological mechanisms between depressive symptoms and metabolic disturbances (Agustini *et al.*, 2020). These findings highlight that women may be more susceptible to the impacts of depression and functional dependence on the accumulation of physical conditions. Regarding age differences, the association between ADL impairment and respiratory-degenerative disease pattern was significant in the 45–60 year age group but not in those over 60. This diminished association in older adults may reflect age-related changes in disease progression, adaptation to chronic conditions, or a plateau effect from already accumulated disease burden. Additionally, other factors such as increased resilience, compensatory behaviours or greater access to healthcare and support services in later life may further weaken the link between functional dependence and subsequent respiratory disease accumulation. Future research should explore these speculations to inform more precise and effective interventions.

The findings have health and clinical implications. Integrating mental health screening – particularly for depression – into routine care of patients with multiple chronic conditions is essential, especially for those with respiratory-degenerative conditions (e.g., chronic lung diseases and arthritis). Individuals with depression should also be monitored for the onset of new physical comorbidities and declines in functional capacity. To break the cycle between multimorbidity and depressive symptoms, coordinated care models that integrate physical, cognitive and mental health services are recommended. Expanding rehabilitation programs to maintain or improve ADL function may help preserve independence and reduce burdens of mental–physical multimorbidity. For instance, occupational therapy interventions (e.g., physical exercises and home-based programs) have demonstrated moderate benefits for ADL performance in older adults (Liu *et al.*, 2018). Incorporating psychological interventions, such as behavioural activation and cognitive-behavioural therapy, as well as mobilizing community resources and strengthening social support networks, can further reduce isolation and promote engagement in meaningful activities, supporting functional independence and mental well-being. Future interventions should consider sex- and age-specific pathways to maximize their effectiveness and address the unique needs of diverse older adult populations.

The strength of this study was that we used four-wave panel data from a nationally representative sample of Chinese adults to clarify the reciprocal relationship of multimorbidity, functional dependence, and depressive symptoms. The use of CLPM models with mediation analysis has the advantage of investigating the dynamic and simultaneous process in which how multimorbidity and depressive symptoms influenced each other, with greater accuracy than independent models (Wei *et al.*, 2019). Second, we measured multimorbidity as both the number of chronic conditions and patterns that capture the interaction among different chronic conditions, which provides a deeper understanding of the impacts of multimorbidity. Third, we adjusted for many confounding variables, including sociodemographic and lifestyle characteristics. The sensitivity analysis also supported the robustness of findings.

Our study also had limitations. First, we used self-reported physician diagnoses or medical treatments to measure multimorbidity, which may underestimate the multimorbidity prevalence as some participants, especially those in rural areas, might have limited access to the healthcare system for timely diagnosis. Second, the numbers and types of chronic conditions measured in CHARLS are comprehensive though not exhaustive, and the severity of conditions was not taken into consideration in this study. Third, our measure of functional dependence was restricted to basic ADLs. While basic ADLs represent a severe and core form of functional disability that is highly relevant to the progression of multimorbidity and depression, they do not capture limitations in instrumental ADLs (IADL). IADL disability often precedes ADL decline and may be more sensitive to early functional and cognitive changes. However, we should note that IADLs may be influenced by a wider array of factors, including socioeconomic status, education, cultural roles and environmental supports, which may introduce more confounding variability. Future studies should incorporate both ADL and IADL measures to provide a more comprehensive picture of how different levels of functional impairment mediate the interplay between physical and mental health. Lastly, the findings might have been affected by selective attrition. However, this concern has been diminished through the inclusion of participants with incomplete data by using reliable methods dealing with missing data and sensitivity analysis.

## Conclusions

This large-scale cohort study uncovered a bi-directional relationship between the number and specific patterns of multimorbidity (i.e., cardiometabolic and respiratory-degenerative) and depressive symptoms among Chinese middle-to-old aged adults, with multimorbidity exerting a stronger effect on later depressive symptoms relative to the predictivity of depressive symptoms on the accumulation of chronic conditions. Notably, the bi-directional association with depressive symptoms was stronger for respiratory-degenerative diseases compared to that with cardiometabolic diseases. The study further identified functional dependence as a partial mediator in the reciprocal associations between multimorbidity and depressive symptoms among both males and females, with a more pronounced mediation effect observed in the context of cardiometabolic diseases. These findings significantly contribute to the extant knowledge on the intricate interplay between multimorbidity, functional dependence, and depressive symptoms within aging populations. Integrated care models should be considered to break the cycle between mental and physical diseases in order to improve patient outcomes. Future studies are warranted to investigate whether interventions to reduce the development and progression of functional dependence could effectively lower the burden of mental and physical multimorbidity.

## Supporting information

10.1017/S2045796026100626.sm001 She et al. supplementary material She et al. supplementary material

## Data Availability

The dataset used in the current study is provided as supplementary material. Additional information is available at https://charls.pku.edu.cn/en/

## Supplementary material

The supplementary material for this article can be found at https://doi.org/10.1017/S2045796026100626
