# Supplementary material for: The interplay of multimorbidity and depressive symptoms: mediation role of functional dependence
Source: Epidemiol Psychiatr Sci. 2026 Apr 7;35:e21. doi: 10.1017/S2045796026100626 (PMC13122540; doi:10.1017/S2045796026100626)
Supplement: She et al. supplementary material [file S2045796026100626sup001.zip › S2045796026100626sup001/SupplementaryMaterials_revised.docx]

**The interplay of multimorbidity and depressive symptoms: mediation role of functional dependence**

| **Supplementary tables** | **Page** |
| --- | --- |
| Supplementary Table S1. Indirect effect estimates of functional dependence in ADL in the reciprocal associations between physical multimorbidity and depressive symptoms from the cross-lagged panel models with mediation among participants with baseline and at least one follow-up data (*n*=15,875) | 4 |
| Supplementary Table S2. Indirect effect estimates of functional dependence in ADL in the reciprocal associations between physical multimorbidity (12 diseases with memory disease excluded) and depressive symptoms from the cross-lagged panel models with mediation among participants with complete data on follow-up surveys (*n*=11,572) | 5 |
| Supplementary Table S3. Correlations of physical multimorbidity measures, functional dependence in ADL and depressive symptoms over four waves of surveys | 6 |
| Supplementary Table S4. Model fit statistics of each of the cross-lagged panel mediation models | 7 |
|  |  |
| **Supplementary figures** |  |
| Supplementary Figure S1. Flowchart of study sample and longitudinal follow-up schedule | 8 |
| Supplementary Figure S2. Repeated Cross-lagged panel models for the mediation effects of functional dependence in activities in daily living (ADL) in the reciprocal associations between condition count and depressive symptoms among participants with both baseline and the at least one follow-up data (*n*=15,875) | 9 |
| Supplementary Figure S3. Repeated cross-lagged panel models for the mediation effects of functional dependence in activities in daily living (ADL) in the reciprocal association between cardiometabolic disease pattern (factor 1) and depressive symptoms among participants with both baseline and at least one follow-up data (*n*=15,875) | 10 |
| Supplementary Figure S4. Repeated cross-lagged panel models for the mediation effects of functional dependence in activities in daily living (ADL) in the reciprocal association between respiratory-degenerative disease pattern (factor 2) and depressive symptoms among participants with both baseline and at least one follow-up data (*n*=15,875) | 11 |
| Supplementary Figure S5. Repeated cross-lagged panel models for the mediation effects of functional dependence in activities in daily living (ADL) in the reciprocal association between condition count (12 conditions with memory disease excluded) and depressive symptoms from 2011 to 2018 (*n*=11,572) | 12 |
| Supplementary Figure S6. Repeated cross-lagged panel models for the mediation effects of functional dependence in activities in daily living (ADL) in the reciprocal association between cardiometabolic disease pattern (factor 1 derived from the 12-condition definition of physical multimorbidity) and depressive symptoms from 2011 to 2018 (*n*=11,572) | 13 |
| Supplementary Figure S7. Repeated cross-lagged panel models for the mediation effects of functional dependence in activities in daily living (ADL) in the reciprocal association between respiratory-degenerative disease pattern (factor 2 derived from the 12-condition definition of physical multimorbidity) and depressive symptoms from 2011 to 2018 (*n*=11,572) | 14 |
| Supplementary Figure S8. Cross-lagged panel models for the mediation effects of functional dependence activities in daily living (ADL) in the reciprocal associations between condition count and depressive symptoms from 2011 to 2018 among males (*n*=5,457) | 15 |
| Supplementary Figure S9. Cross-lagged panel models for the mediation effects of functional dependence in activities in daily living (ADL) in the reciprocal associations between cardiometabolic disease pattern (factor 1) and depressive symptoms from 2011 to 2018 among males (*n*=5,457) | 16 |
| Supplementary Figure S10. Cross-lagged panel models for the mediation effects of functional dependence in activities in daily living (ADL) in the reciprocal associations between respiratory-degenerative disease pattern (factor 2) and depressive symptoms from 2011 to 2018 among males (*n*=5,457) | 17 |
| Supplementary Figure S11. Cross-lagged panel models for the mediation effects of functional dependence in in activities in daily living (ADL) in the reciprocal associations between condition count and depressive symptoms from 2011 to 2018 among females (*n*=6,115) | 18 |
| Supplementary Figure S12. Cross-lagged panel models for the mediation effects of functional dependence in activities in daily living (ADL) in the reciprocal associations between cardiometabolic disease pattern (factor 1) and depressive symptoms from 2011 to 2018 among females (*n*=6,115) | 19 |
| Supplementary Figure S13. Cross-lagged panel models for the mediation effects of functional dependence in activities in daily living (ADL) in the reciprocal associations between respiratory-degenerative disease pattern (factor 2) and depressive symptoms from 2011 to 2018 among females (*n*=6,115) | 20 |
| Supplementary Figure S14. Cross-lagged panel models for the mediation effects of functional dependence activities in daily living (ADL) in the reciprocal associations between condition count and depressive symptoms from 2011 to 2018 among age of 60 or less (n=7,341). | 21 |
| Supplementary Figure S15. Cross-lagged panel models for the mediation effects of functional dependence activities in daily living (ADL) in the reciprocal associations between cardiometabolic disease pattern (factor 1) and depressive symptoms from 2011 to 2018 among age of 60 or less (n=7,341). | 22 |
| Supplementary Figure S16. Cross-lagged panel models for the mediation effects of functional dependence activities in daily living (ADL) in the reciprocal associations between respiratory-degenerative disease pattern (factor 2) and depressive symptoms from 2011 to 2018 among age of 60 or less (n=7,341). | 23 |
| Supplementary Figure S17. Cross-lagged panel models for the mediation effects of functional dependence activities in daily living (ADL) in the reciprocal associations between condition count and depressive symptoms from 2011 to 2018 among beyond age of 60 (n=4,214). | 24 |
| Supplementary Figure S18. Cross-lagged panel models for the mediation effects of functional dependence activities in daily living (ADL) in the reciprocal associations between cardiometabolic disease pattern (factor 1) and depressive symptoms from 2011 to 2018 among beyond age of 60 (n=4,214). | 25 |
| Supplementary Figure S19. Cross-lagged panel models for the mediation effects of functional dependence activities in daily living (ADL) in the reciprocal associations between respiratory-degenerative disease pattern (factor 2) and depressive symptoms from 2011 to 2018 among beyond age of 60 (n=4,214). | 26 |

**Supplementary Table S1.** Indirect effect estimates of functional dependence in ADL in the reciprocal associations between physical multimorbidity and depressive symptoms from the cross-lagged panel models with mediation among participants with baseline and at least one follow-up data (*n*=15,875)

*Note:* CI, confidence interval; ADL, functional dependence in activities in daily living (ADL).

| **Paths** | **Standardized indirect effect**  ***β* (95% CI)** | **Proportion mediated**  **(%)** |
| --- | --- | --- |
| **Chronic condition count** |  |  |
| Depressive symptoms (T1) → ADL (T2)→ Condition count (T3) | 0.003 [0.003, 0.005] | 6.4 |
| Condition count (T1) → ADL (T2)→ Depressive symptoms (T3) | 0.012 [0.009, 0.015] | 7.7 |
| Depressive symptoms (T2) → ADL (T3)→ Condition count (T4) | 0.004 [0.003, 0.005] | 7.1 |
| Condition count (T2) → ADL (T3)→ Depressive symptoms (T4) | 0.008 [0.006, 0.011] | 5.9 |
| **Cardiometabolic disease pattern (Factor 1)** |  |  |
| Depressive symptoms (T1) → ADL (T2) → Factor 1 score (T3) | 0.004 [0.002, 0.005] | 18.5 |
| Factor 1 score (T1) → ADL (T2)→ Depressive symptoms (T3) | 0.011 [0.009, 0.014] | 8.9 |
| Depressive symptoms (T2) → ADL (T3)→ Factor 1 score (T4) | 0.005 [0.003, 0.007] | 11.7 |
| Factor 1 score (T2) → ADL (T3)→ Depressive symptoms (T4) | 0.009 [0.007, 0.011] | 10.4 |
| **Respiratory-degenerative diseases pattern (Factor 2)** |  |  |
| Depressive symptoms (T1)→ ADL (T2)→ Factor 2 score (T3) | 0.002 [0.001, 0.003] | 7.0 |
| Factor 2 score (T1) → ADL (T2)→ Depressive symptoms (T3) | 0.009 [0.007, 0.012] | 6.9 |
| Depressive symptoms (T2) → ADL (T3)→ Factor 2 score (T4) | 0.005 [0.003, 0.008] | 5.6 |
| Factor 2 score (T2) → ADL (T3)→ Depressive symptoms (T4) | 0.007 [0.005, 0.009] | 5.2 |

**Supplementary Table S2.** Indirect effect estimates of functional dependence in ADL in the reciprocal associations between physical multimorbidity and depressive symptoms from the cross-lagged panel models with mediation among participants with complete data on follow-up surveys (*n*=11,572)

*Note:* CI, confidence interval; ADL, functional dependence in activities in daily living (ADL).

| **Paths** | **Standardized indirect effect**  ***β* (95% CI)** | **Proportion mediated**  **(%)** |
| --- | --- | --- |
| **Chronic condition count** |  |  |
| Depressive symptoms (T1) → ADL (T2)→ Condition count (T3) | 0.003 [0.002, 0.005] | 7.3 |
| Condition count (T1) → ADL (T2)→ Depressive symptoms (T3) | 0.010 [0.007, 0.014] | 5.8 |
| Depressive symptoms (T2) → ADL (T3)→ Condition count (T4) | 0.005 [0.003, 0.006] | 7.3 |
| Condition count (T2) → ADL (T3)→ Depressive symptoms (T4) | 0.009 [0.006, 0.012] | 6.1 |
| **Cardiometabolic disease pattern (Factor 1)** |  |  |
| Depressive symptoms (T1) → ADL (T2) → Factor 1 score (T3) | 0.003 [0.001, 0.004] | 20.2 |
| Factor 1 score (T1) → ADL (T2)→ Depressive symptoms (T3) | 0.009 [0.006, 0.011] | 9.5 |
| Depressive symptoms (T2) → ADL (T3)→ Factor 1 score (T4) | 0.004 [0.002, 0.005] | 11.23 |
| Factor 1 score (T2) → ADL (T3)→ Depressive symptoms (T4) | 0.007 [0.005, 0.010] | 9.9 |
| **Respiratory-degenerative diseases pattern (Factor 2)** |  |  |
| Depressive symptoms (T1)→ ADL (T2)→ Factor 2 score (T3) | 0.001 [0.000, 0.003] | 4.1 |
| Factor 2 score (T1) → ADL (T2)→ Depressive symptoms (T3) | 0.008 [0.005, 0.011] | 5.6 |
| Depressive symptoms (T2) → ADL (T3)→ Factor 2 score (T4) | 0.003 [0.001, 0.004] | 4.9 |
| Factor 2 score (T2) → ADL (T3)→ Depressive symptoms (T4) | 0.007 [0.004, 0.010] | 5.2 |

**Supplementary Table S3.** Correlations of physical multimorbidity measures, functional dependence in ADL and depressive symptoms over four waves of surveys

|  | **1** | **2** | **3** | **4** | **5** | **6** | **7** | **8** | **9** | **10** | **11** | **12** | **13** | **14** | **15** | **16** | **17** | **18** | **19** | **20** |
| --- | --- | --- | --- | --- | --- | --- | --- | --- | --- | --- | --- | --- | --- | --- | --- | --- | --- | --- | --- | --- |
| 1. T1 Condition count | 1.00 |  |  |  |  |  |  |  |  |  |  |  |  |  |  |  |  |  |  |  |
| 2. T1 Factor 1 | .78^**^ | 1.00 |  |  |  |  |  |  |  |  |  |  |  |  |  |  |  |  |  |  |
| 3. T1 Factor 2 | .69^**^ | .15^**^ | 1.00 |  |  |  |  |  |  |  |  |  |  |  |  |  |  |  |  |  |
| 4. T1 CESD | .34^**^ | .17^**^ | .30^**^ | 1.00 |  |  |  |  |  |  |  |  |  |  |  |  |  |  |  |  |
| 5. T1 ADL | .26^**^ | .17^**^ | .19^**^ | .31^**^ | 1.00 |  |  |  |  |  |  |  |  |  |  |  |  |  |  |  |
| 6. T2 Condition count | .81^**^ | .68^**^ | .55^**^ | .31^**^ | .24^**^ | 1.00 |  |  |  |  |  |  |  |  |  |  |  |  |  |  |
| 7. T2 Factor 1 | .66^**^ | .87^**^ | .12^**^ | .16^**^ | .17^**^ | .79^**^ | 1.00 |  |  |  |  |  |  |  |  |  |  |  |  |  |
| 8. T2 Factor 2 | .57^**^ | .12^**^ | .85^**^ | .29^**^ | .17^**^ | .68^**^ | .15^**^ | 1.00 |  |  |  |  |  |  |  |  |  |  |  |  |
| 9. T2 CESD | .28^**^ | .14^**^ | .25^**^ | .50^**^ | .19^**^ | .33^**^ | .18^**^ | .28^**^ | 1.00 |  |  |  |  |  |  |  |  |  |  |  |
| 10. T2 ADL | .25^**^ | .17^**^ | .19^**^ | .24^**^ | .39^**^ | .27^**^ | .19^**^ | .20^**^ | .29^**^ | 1.00 |  |  |  |  |  |  |  |  |  |  |
| 11. T3 Condition count | .79^**^ | .65^**^ | .54^**^ | .32^**^ | .24^**^ | .84^**^ | .70^**^ | .57^**^ | .32^**^ | .26^**^ | 1.00 |  |  |  |  |  |  |  |  |  |
| 12. T3 Factor 1 | .63^**^ | .82^**^ | .12^**^ | .16^**^ | .16^**^ | .69^**^ | .88^**^ | .13^**^ | .18^**^ | .19^**^ | .80^**^ | 1.00 |  |  |  |  |  |  |  |  |
| 13. T3 Factor 2 | .55^**^ | .13^**^ | .81^**^ | .29^**^ | .17^**^ | .57^**^ | .13^**^ | .86^**^ | .27^**^ | .19^**^ | .67^**^ | .14^**^ | 1.00 |  |  |  |  |  |  |  |
| 14. T3 CESD | .28^**^ | .15^**^ | .23^**^ | .48^**^ | .20^**^ | .30^**^ | .17^**^ | .25^**^ | .53^**^ | .24^**^ | .34^**^ | .19^**^ | .27^**^ | 1.00 |  |  |  |  |  |  |
| 15. T3 ADL | .26^**^ | .19^**^ | .19^**^ | .26^**^ | .42^**^ | .27^**^ | .18^**^ | .20^**^ | .25^**^ | .49^**^ | .30^**^ | .21^**^ | .21^**^ | .34^**^ | 1.00 |  |  |  |  |  |
| 16. T4 Condition count | .69^**^ | .56^**^ | .49^**^ | .31^**^ | .21^**^ | .71^**^ | .58^**^ | .49^**^ | .31^**^ | .25^**^ | .78^**^ | .65^**^ | .52^**^ | .33^**^ | .30^**^ | 1.00 |  |  |  |  |
| 17. T4 Factor 1 | .55^**^ | .68^**^ | .14^**^ | .17^**^ | .15^**^ | .58^**^ | .71^**^ | .15^**^ | .18^**^ | .17^**^ | .64^**^ | .80^**^ | .14^**^ | .20^**^ | .22^**^ | .81^**^ | 1.00 |  |  |  |
| 18. T4 Factor 2 | .50^**^ | .14^**^ | .70^**^ | .27^**^ | .16^**^ | .49^**^ | .14^**^ | .70^**^ | .27^**^ | .18^**^ | .53^**^ | .14^**^ | .76^**^ | .28^**^ | .21^**^ | .69^**^ | .19^**^ | 1.00 |  |  |
| 19. T4 CESD | .24^**^ | .12^**^ | .22^**^ | .42^**^ | .17^**^ | .25^**^ | .13^**^ | .23^**^ | .48^**^ | .21^**^ | .27^**^ | .14^**^ | .24^**^ | .51^**^ | .25^**^ | .32^**^ | .19^**^ | .27^**^ | 1.00 |  |
| 20. T4 ADL | .26^**^ | .19^**^ | .19^**^ | .23^**^ | .36^**^ | .26^**^ | .18^**^ | .20^**^ | .22^**^ | .44^**^ | .26^**^ | .19^**^ | .19^**^ | .25^**^ | .52^**^ | .32^**^ | .25^**^ | .22^**^ | .31^**^ | 1.00 |
| Mean | 1.94 | 0.18 | 0.18 | 8.39 | 0.31 | 2.12 | 0.21 | 0.18 | 7.92 | 0.31 | 2.49 | 0.26 | 0.22 | 8.13 | 0.43 | 2.83 | 0.32 | 0.23 | 8.78 | 0.52 |
| SD | 1.64 | 0.22 | 0.22 | 6.29 | 0.89 | 1.72 | 0.23 | 0.23 | 5.76 | 0.87 | 1.88 | 0.26 | 0.25 | 6.41 | 1.04 | 2.10 | 0.29 | 0.28 | 6.54 | 1.21 |

*Note:* Factor 1, cardiometabolic diseases pattern; Factor 2, respiratory-degenerative diseases pattern; CESD, depressive symptoms assessed by the 10‐item Center for Epidemiologic Studies Depression Scale (CESD-10); ADL, functional dependence in activities in daily living (ADL).

** *p*<0.01

**Supplementary Table S4.** Model fit statistics of each of the cross-lagged panel mediation models

| **Models** | **CFI** | **SRMR** | **RMSEA (90% CI)** |
| --- | --- | --- | --- |
| Condition count model | 0.915 | 0.051 | 0.056 (0.055, 0.058) |
| Factor 1 model (cardiometabolic disease) | 0.930 | 0.052 | 0.052 (0.051, 0.053) |
| Factor 2 model (respiratory-degenerative disease) | 0.915 | 0.052 | 0.056 (0.055, 0.058) |

*Note:* CFI, comparative fit index; SRMR, standardized root mean square residual; RMSEA, root mean square error of approximation; CI, confidence interval.


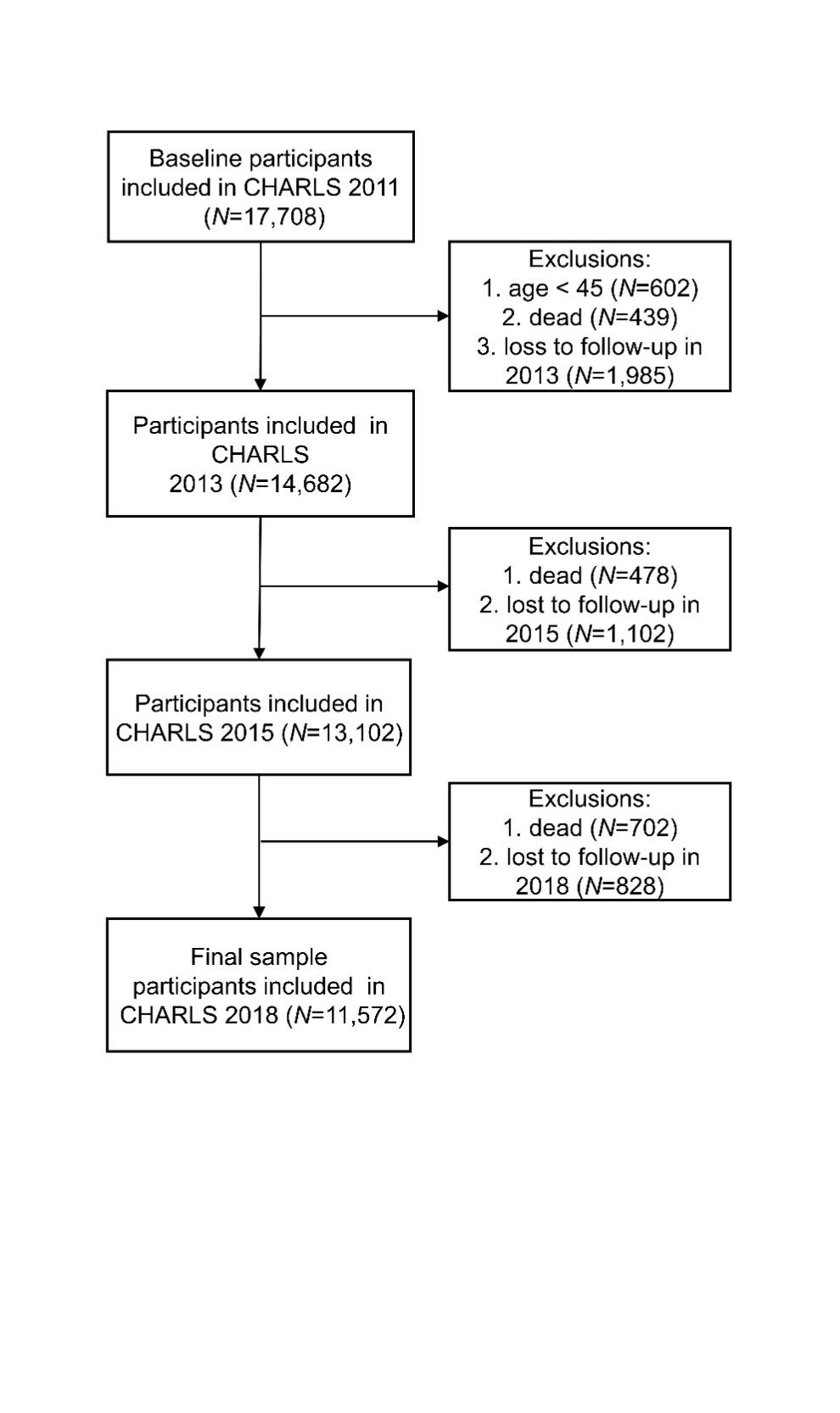


**Supplementary Figure S1.** Flowchart of study sample and longitudinal follow-up schedule

**
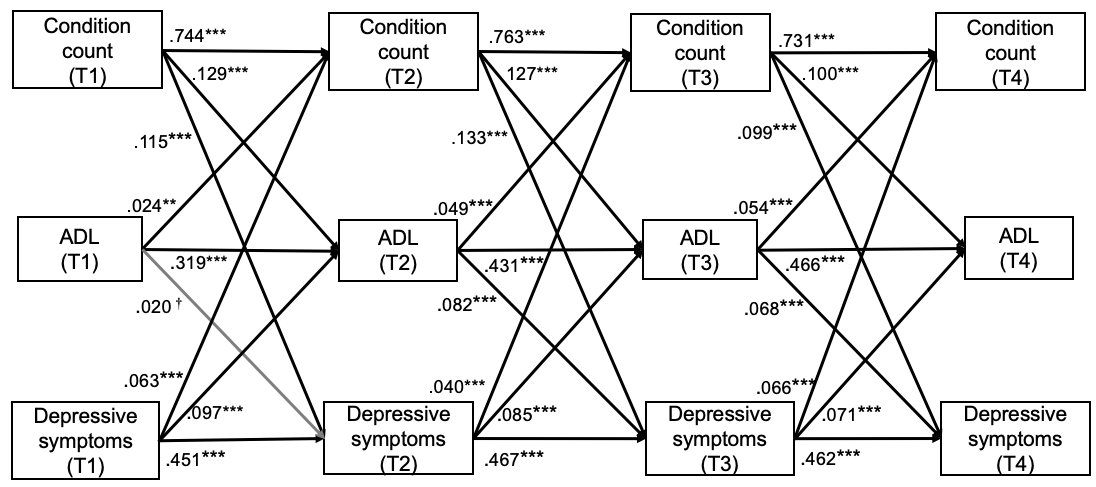
**

**Supplementary Figure S2.** Repeated cross-lagged panel models for the mediation effects of functional dependence in activities in daily living (ADL) in the reciprocal associations between condition count and depressive symptoms among participants with both baseline and at least one follow-up data (*n*=15,875). Solid lines represent the significance of the structural path (*p*< 0.05) while dash lines represent non-significant paths, and grey lines represent marginal significant paths (0.05<*p* <0.10). Standardized coefficients were shown. For simplicity, covariates of outcomes and correlation paths are not presented. ****p* <0.001, ***p* <0.01, **p* <0.05, **^†^**0.05<*p* <0.10.


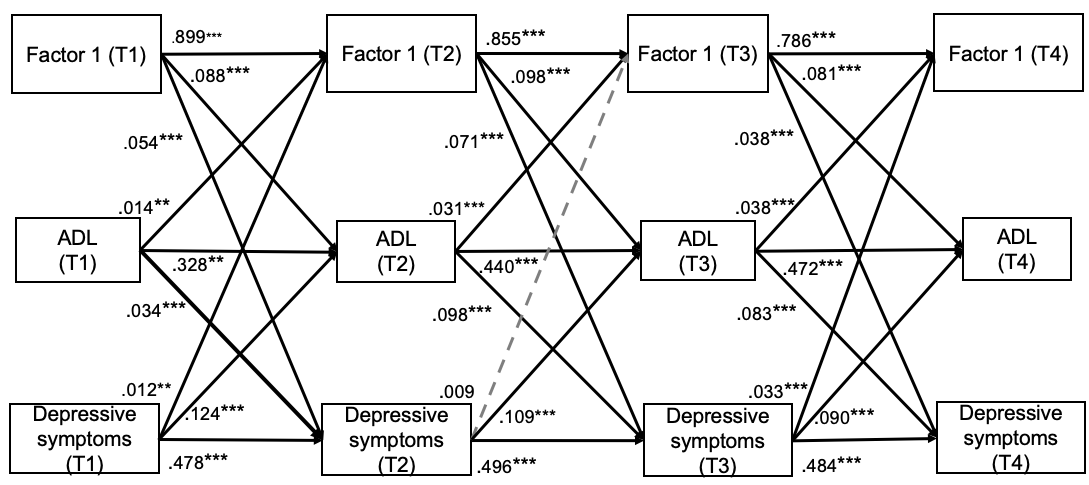


**Supplementary Figure S3.** Repeated cross-lagged panel models for the mediation effects of functional dependence in activities in daily living (ADL) in the reciprocal association between cardiometabolic disease pattern (factor 1) and depressive symptoms among participants with both baseline and at least one follow-up data (*n*=15,875). Solid lines represent the significance of the structural path (*p*< 0.05) while dash lines represent non-significant paths, and grey lines represent marginal significant paths (0.05<*p* <0.10). Standardized coefficients were shown. For simplicity, covariates of outcomes and correlation paths are not presented. ****p* <0.001, ***p* <0.01, **p* <0.05, **^†^**0.05<*p* <0.10.


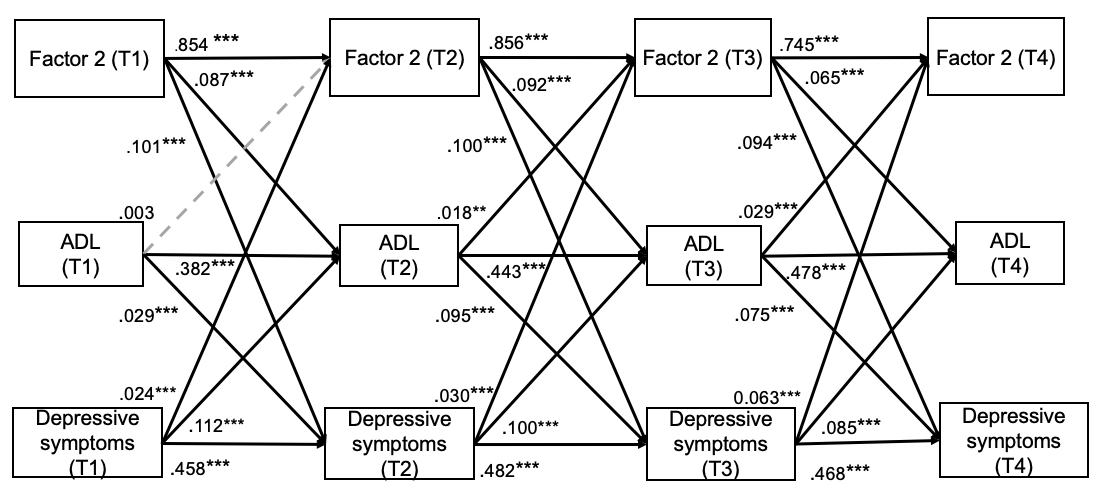


**Supplementary Figure S4.** Repeated cross-lagged panel models for the mediation effects of functional dependence in activities in daily living (ADL) in the reciprocal association between respiratory-degenerative disease pattern (factor 2) and depressive symptoms among participants with both baseline and at least one follow-up data (*n*=15,875). Solid lines represent the significance of the structural path (*p*< 0.05) while dash lines represent non-significant paths, and grey lines represent marginal significant paths (0.05<*p* <0.10). Standardized coefficients were shown. For simplicity, covariates of outcomes and correlation paths are not presented. ****p* <0.001, ***p* <0.01, **p* <0.05, **^†^**0.05<*p* <0.10.


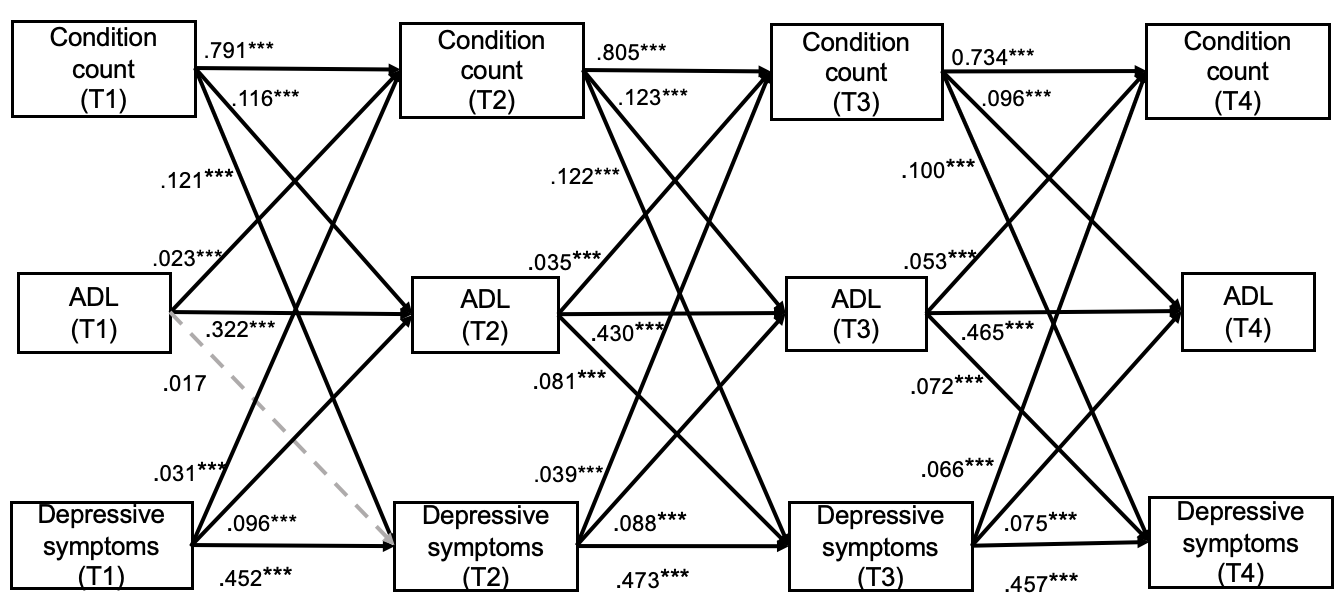


**Supplementary Figure S5.** Repeated cross-lagged panel models for the mediation effects of functional dependence in activities in daily living (ADL) in the reciprocal association between condition count (12 conditions with memory disease excluded) and depressive symptoms from 2011 to 2018 (*n*=11,572)**.** Solid lines represent the significance of the structural path (*p*< 0.05) while dash lines represent non-significant paths, and grey lines represent marginal significant paths (0.05<*p* <0.10). Standardized coefficients were shown. For simplicity, covariates of outcomes and correlation paths are not presented. ****p* <0.001, ***p* <0.01, **p* <0.05, **^†^**0.05<*p* <0.10.


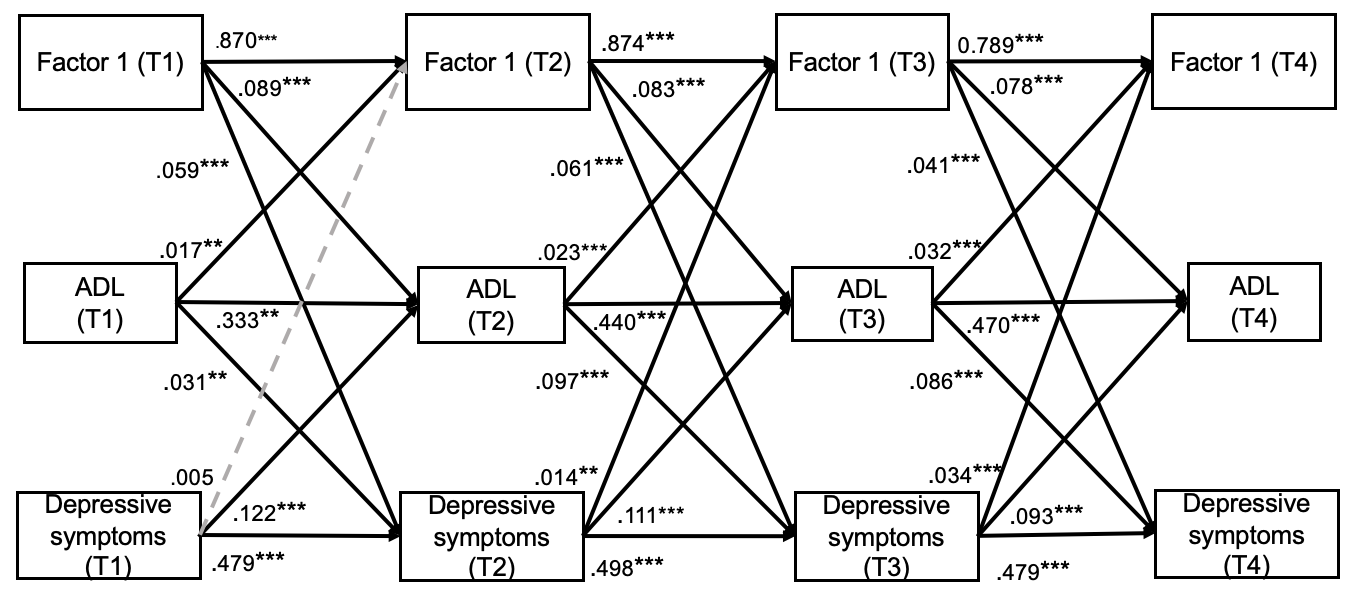


**Supplementary Figure S6.** Repeated cross-lagged panel models for the mediation effects of functional dependence in activities in daily living (ADL) in the reciprocal association between cardiometabolic disease pattern (factor 1) derived from the 12-condition definition of physical multimorbidity) and depressive symptoms from 2011 to 2018 (*n*=11,572)**.** Solid lines represent the significance of the structural path (*p*< 0.05) while dash lines represent non-significant paths, and grey lines represent marginal significant paths (0.05<*p* <0.10). Standardized coefficients were shown. For simplicity, covariates of outcomes and correlation paths are not presented. ****p* <0.001, ***p* <0.01, **p* <0.05, **^†^**0.05<*p* <0.10.


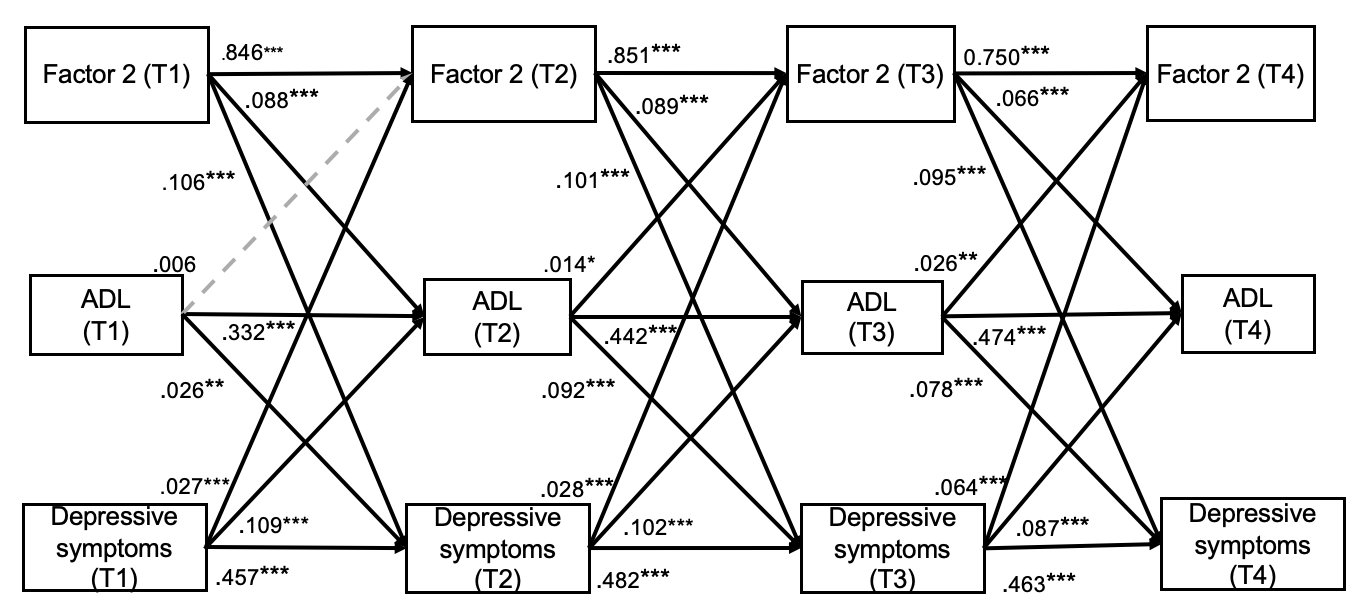


**Supplementary Figure S7.** Repeated cross-lagged panel models for the mediation effects of functional dependence in activities in daily living (ADL) in the reciprocal association between respiratory-degenerative disease pattern (factor 2) derived from the 12-condition definition of physical multimorbidity) and depressive symptoms from 2011 to 2018 (*n*=11,572)**.** Solid lines represent the significance of the structural path (*p*< 0.05) while dash lines represent non-significant paths, and grey lines represent marginal significant paths (0.05<*p* <0.10). Standardized coefficients were shown. For simplicity, covariates of outcomes and correlation paths are not presented. ****p* <0.001, ***p* <0.01, **p* <0.05, **^†^**0.05<*p* <0.10.

**
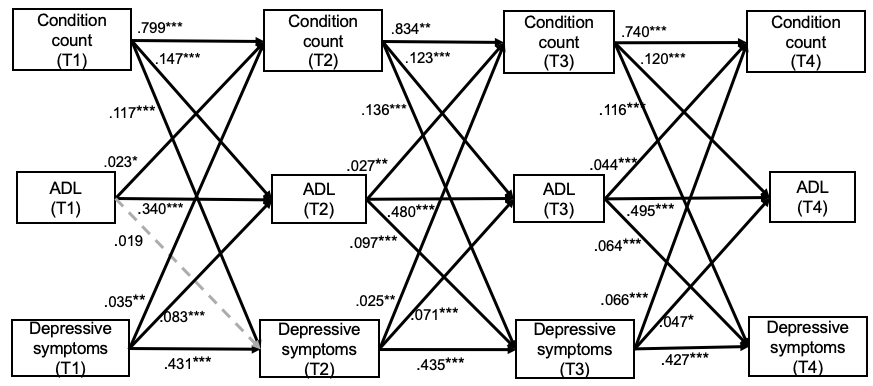
**

**Supplementary Figure S8.** Cross-lagged panel models for the mediation effects of functional dependence activities in daily living (ADL) in the reciprocal associations between condition count and depressive symptoms from 2011 to 2018 among males (*n*=5,457)**.** Solid lines represent the significance of the structural path (*p*< 0.05) while dash lines represent non-significant paths, and grey lines represent marginal significant paths (0.05<*p* <0.10). Standardized coefficients were shown. For simplicity, covariates of outcomes and correlation paths are not presented. ****p* <0.001, ***p* <0.01, **p* <0.05, **^†^**0.05<*p* <0.10.

**
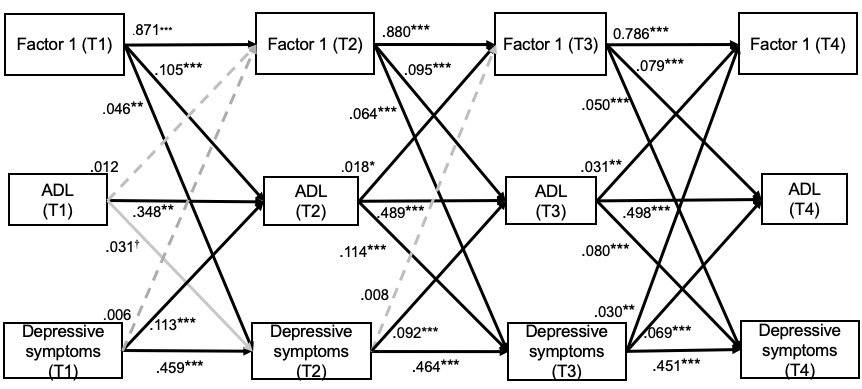
**

**Supplementary Figure S9.** Cross-lagged panel models for the mediation effects of functional dependence in activities in daily living (ADL) in the reciprocal associations between cardiometabolic disease pattern (factor 1) and depressive symptoms from 2011 to 2018 among males (*n*=5,457). Solid lines represent the significance of the structural path (*p*< 0.05) while dash lines represent non-significant paths, and grey lines represent marginal significant paths (0.05<*p* <0.10). Standardized coefficients were shown. For simplicity, covariates of outcomes and correlation paths are not presented. ****p* <0.001, ***p* <0.01, **p* <0.05, **^†^**0.05<*p* <0.10.

**
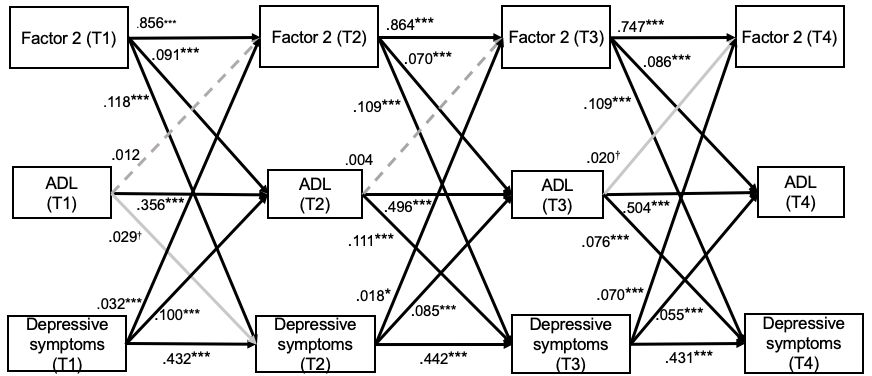
**

**Supplementary Figure S10.** Cross-lagged panel models for the mediation effects of functional dependence in activities in daily living (ADL) in the reciprocal associations between respiratory-degenerative disease pattern (factor 2) and depressive symptoms from 2011 to 2018 among males (*n*=5,457). Solid lines represent the significance of the structural path (*p*< 0.05) while dash lines represent non-significant paths, and grey lines represent marginal significant paths (0.05<*p* <0.10). Standardized coefficients were shown. For simplicity, covariates of outcomes and correlation paths are not presented. ****p* <0.001, ***p* <0.01, **p* <0.05, **^†^**0.05<*p* <0.10.

**
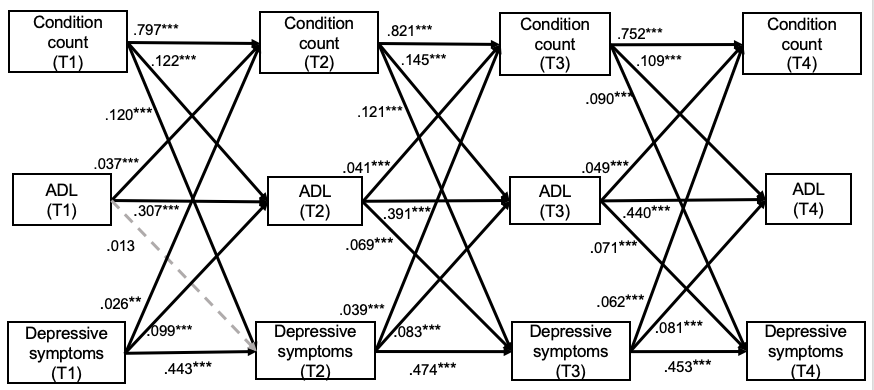
**

**Supplementary Figure S11.** Cross-lagged panel models for the mediation effects of functional dependence in in activities in daily living (ADL) in the reciprocal associations between condition count and depressive symptoms from 2011 to 2018 among females (*n*=6,115)**.** Solid lines represent the significance of the structural path (*p*< 0.05) while dash lines represent non-significant paths, and grey lines represent marginal significant paths (0.05<*p* <0.10). Standardized coefficients were shown. For simplicity, covariates of outcomes and correlation paths are not presented. ****p* <0.001, ***p* <0.01, **p* <0.05, **^†^**0.05<*p* <0.10.

**
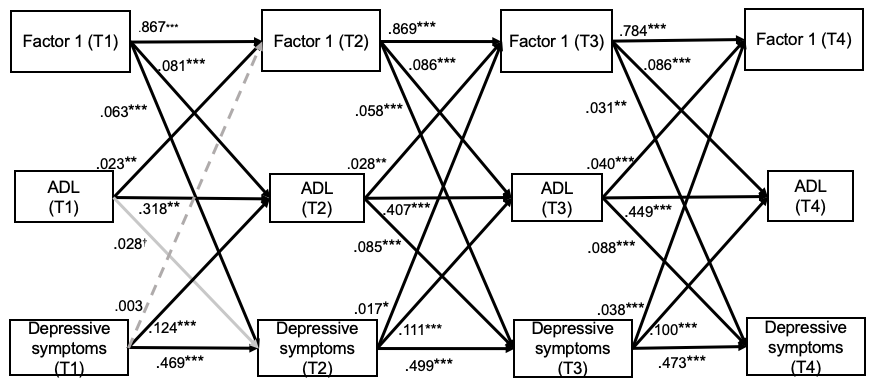
**

**Supplementary Figure S12.** Cross-lagged panel models for the mediation effects of functional dependence in activities in daily living (ADL) in the reciprocal associations between cardiometabolic disease pattern (factor 1) and depressive symptoms from 2011 to 2018 among females (*n*=6,115). Solid lines represent the significance of the structural path (*p*< 0.05) while dash lines represent non-significant paths, and grey lines represent marginal significant paths (0.05<*p* <0.10). Standardized coefficients were shown. For simplicity, covariates of outcomes and correlation paths are not presented. ****p* <0.001, ***p* <0.01, **p* <0.05, **^†^**0.05<*p* <0.10.

**
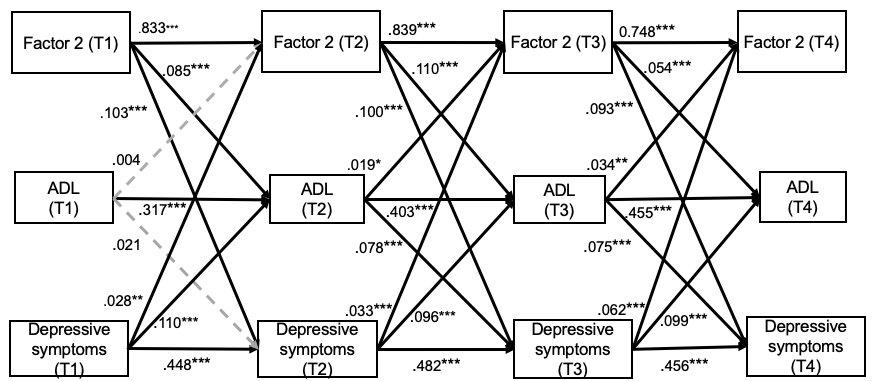
**

**Supplementary Figure S13.** Cross-lagged panel models for the mediation effects of functional dependence in activities in daily living (ADL) in the reciprocal associations between respiratory-degenerative disease pattern (factor 2) and depressive symptoms from 2011 to 2018 among females (*n*=6,115). Solid lines represent the significance of the structural path (*p*< 0.05) while dash lines represent non-significant paths, and grey lines represent marginal significant paths (0.05<*p* <0.10). Standardized coefficients were shown. For simplicity, covariates of outcomes and correlation paths are not presented. ****p* <0.001, ***p* <0.01, **p* <0.05, **^†^**0.05<*p* <0.10.


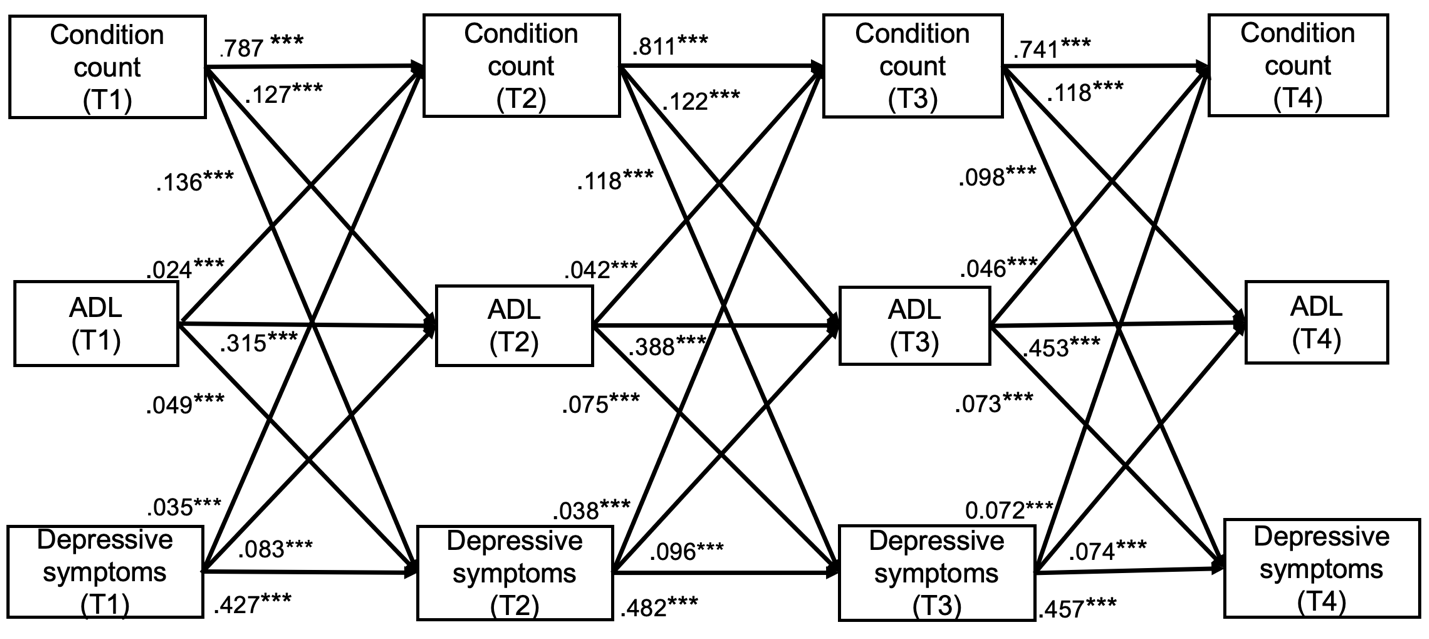


**Supplementary Figure S14**. Cross-lagged panel models for the mediation effects of functional dependence in activities in daily living (ADL) in the reciprocal associations between condition count and depressive symptoms from 2011 to 2018 among pariticipants aged 60 or less (*n*=7,341). Solid lines represent the significance of the structural path (*p*< 0.05) while dash lines represent non-significant paths, and grey lines represent marginal significant paths (0.05<*p*<0.10). Standardized coefficients were shown. For simplicity, covariates of outcomes and correlation paths are not presented. ****p* <0.001, ***p* <0.01, **p* <0.05, †0.05<*p*<0.10.


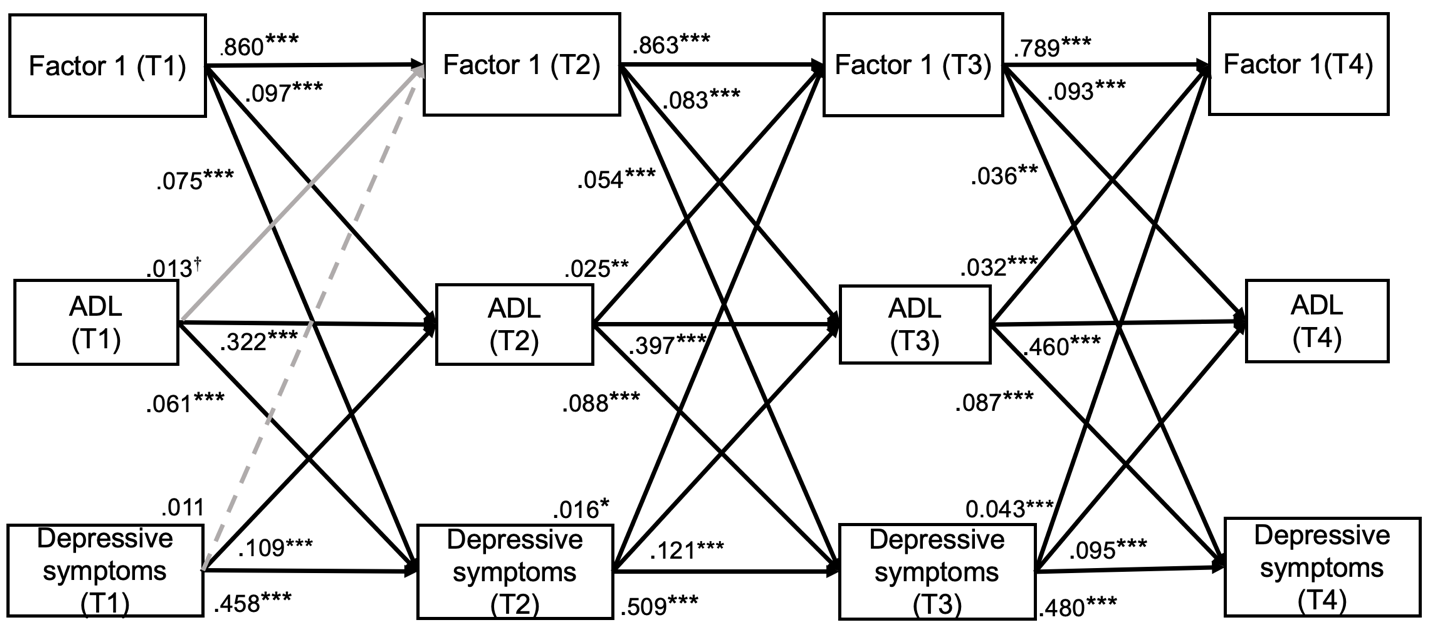


**Supplementary Figure S15**. Cross-lagged panel models for the mediation effects of functional dependence in activities in daily living (ADL) in the reciprocal associations between cardiometabolic disease pattern (factor 1) and depressive symptoms from 2011 to 2018 among pariticipants aged 60 or less (*n*=7,341). Solid lines represent the significance of the structural path (*p*< 0.05) while dash lines represent non-significant paths, and grey lines represent marginal significant paths (0.05<*p*<0.10). Standardized coefficients were shown. For simplicity, covariates of outcomes and correlation paths are not presented. ****p* <0.001, ***p* <0.01, **p* <0.05, †0.05<*p*<0.10.


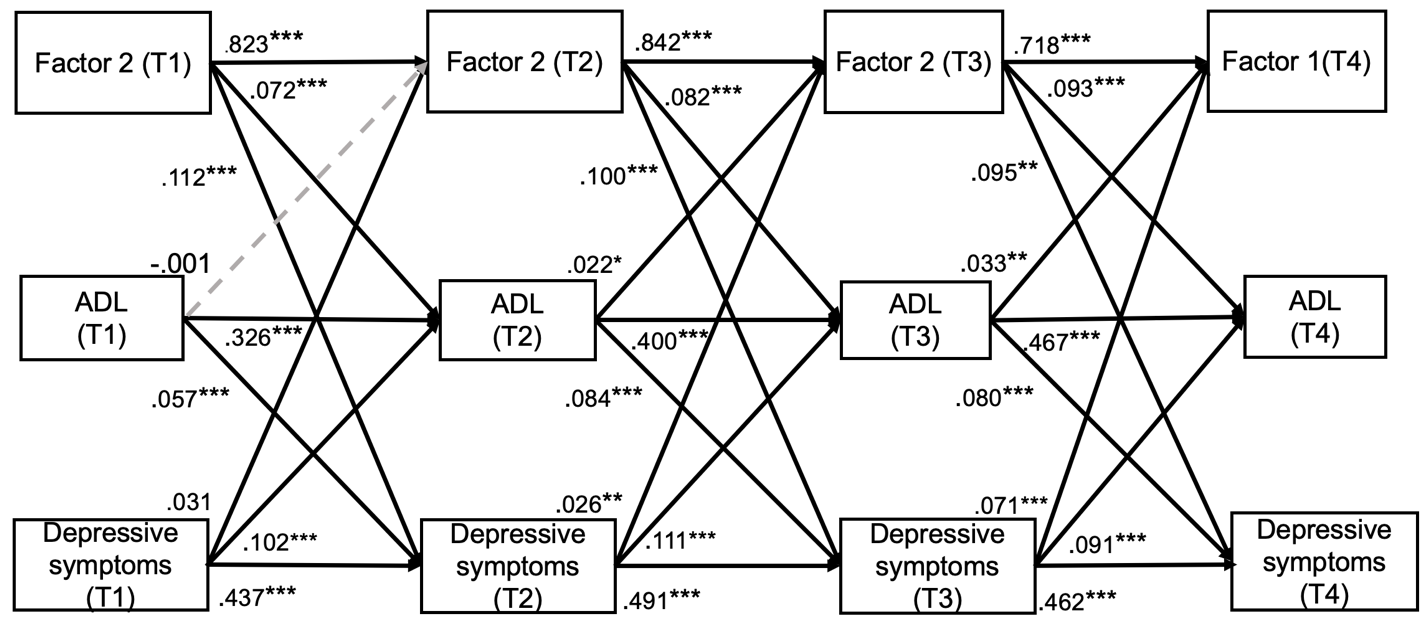


**Supplementary Figure S16**. Cross-lagged panel models for the mediation effects of functional dependence in activities in daily living (ADL) in the reciprocal associations between respiratory-degenerative disease pattern (factor 2) and depressive symptoms from 2011 to 2018 among pariticipants aged 60 or less (*n*=7,341). Solid lines represent the significance of the structural path (*p*< 0.05) while dash lines represent non-significant paths, and grey lines represent marginal significant paths (0.05<*p* <0.10). Standardized coefficients were shown. For simplicity, covariates of outcomes and correlation paths are not presented. ****p* <0.001, ***p* <0.01, **p* <0.05, †0.05<*p*<0.10.


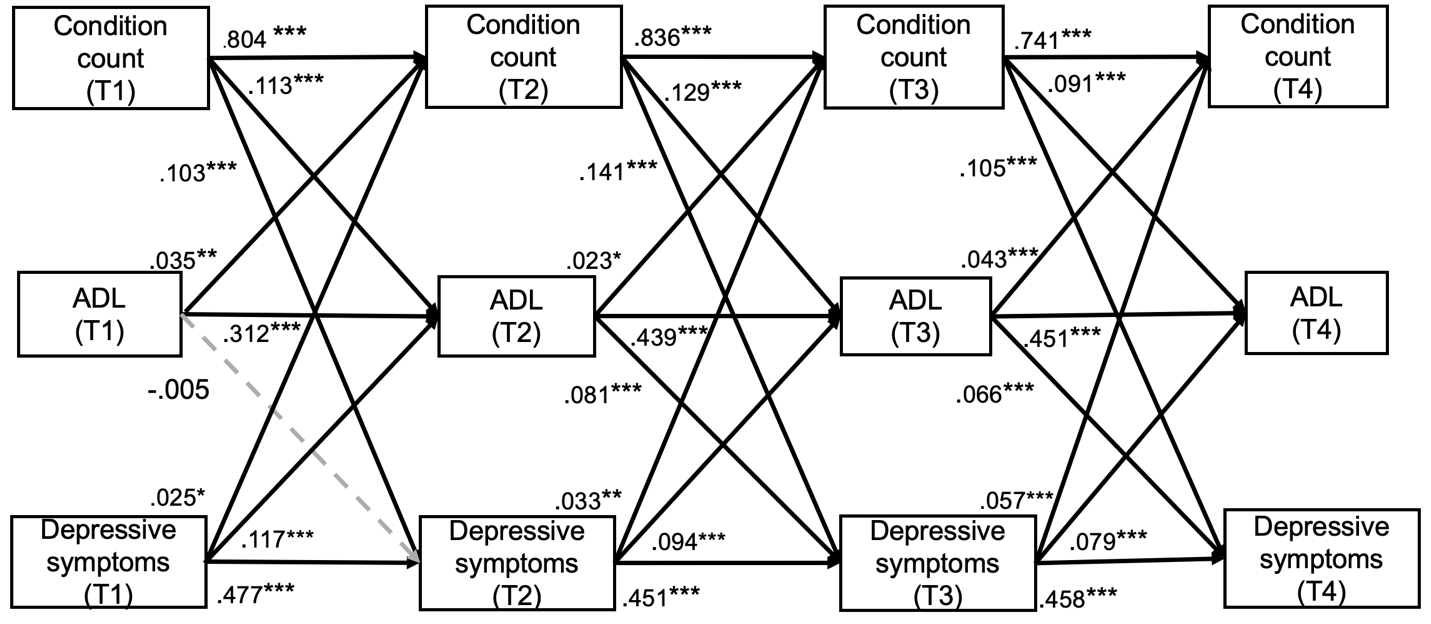


**Supplementary Figure S17**. Cross-lagged panel models for the mediation effects of functional dependence in activities in daily living (ADL) in the reciprocal associations between condition count and depressive symptoms from 2011 to 2018 among pariticipants aged >60 years (*n*=4,214). Solid lines represent the significance of the structural path (*p*<0.05) while dash lines represent non-significant paths, and grey lines represent marginal significant paths (0.05<*p*<0.10). Standardized coefficients were shown. For simplicity, covariates of outcomes and correlation paths are not presented. ****p* <0.001, ***p* <0.01, **p* <0.05, †0.05<*p* <0.10.


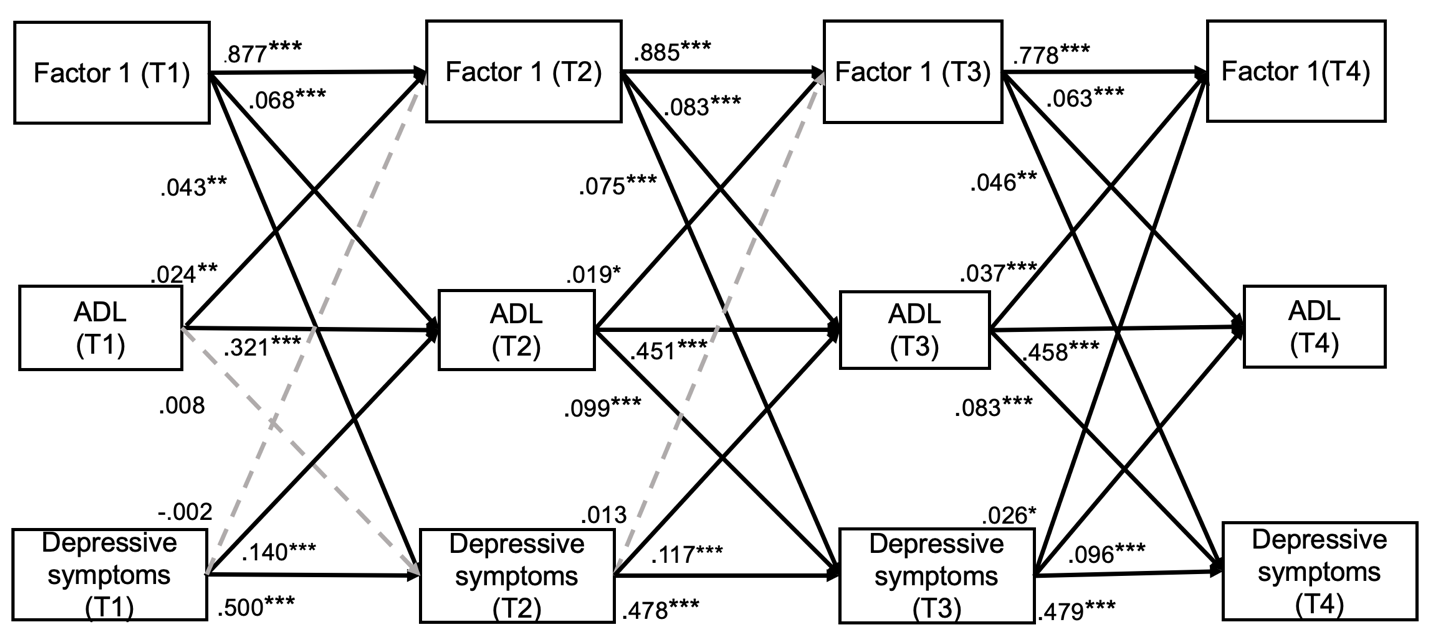


**Supplementary Figure S18**. Cross-lagged panel models for the mediation effects of functional dependence in activities in daily living (ADL) in the reciprocal associations between cardiometabolic disease pattern (factor 1) and depressive symptoms from 2011 to 2018 among pariticipants aged >60 years (*n*=4,214). Solid lines represent the significance of the structural path (*p*< 0.05) while dash lines represent non-significant paths, and grey lines represent marginal significant paths (0.05<*p*<0.10). Standardized coefficients were shown. For simplicity, covariates of outcomes and correlation paths are not presented. ****p* <0.001, ***p* <0.01, **p* <0.05, †0.05<*p*<0.10.


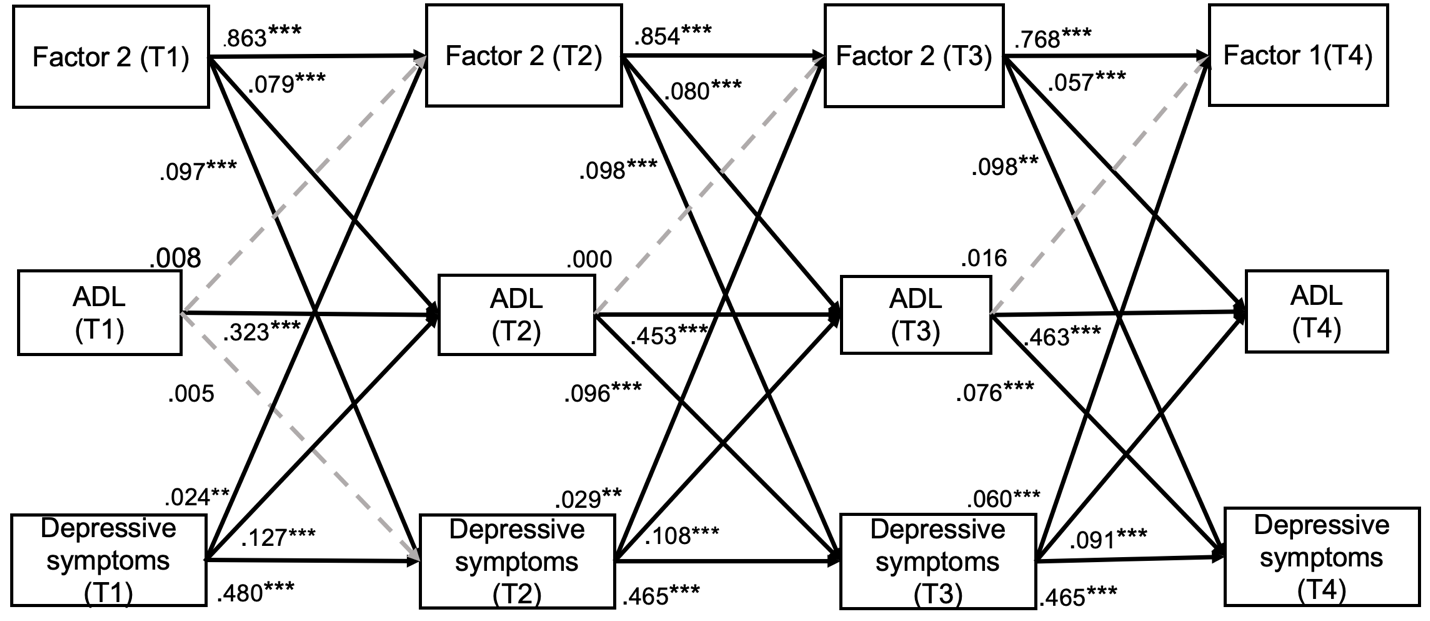


**Supplementary Figure S19**. Cross-lagged panel models for the mediation effects of functional dependence in activities in daily living (ADL) in the reciprocal associations between respiratory-degenerative disease pattern (factor 2) and depressive symptoms from 2011 to 2018 among pariticipants aged >60 years (*n*=4,214). Solid lines represent the significance of the structural path (*p*< 0.05) while dash lines represent non-significant paths, and grey lines represent marginal significant paths (0.05<*p* <0.10). Standardized coefficients were shown. For simplicity, covariates of outcomes and correlation paths are not presented. ****p*<0.001, ***p* <0.01, **p* <0.05, †0.05<*p* <0.10.
